# Supplementary material for: Madurastatins with Imidazolidinone Rings: Natural Products or Side-Reaction Products from Extraction Solvents?
Source: Int J Mol Sci. 2023 Dec 25;25(1):301. doi: 10.3390/ijms25010301 (PMC10779116; doi:10.3390/ijms25010301)
Supplement: Supplementary file 1 [file ijms-25-00301-s001.zip › ijms-2764966-supplementary.pdf]

Supplementary information for

Madurastatins with Imidazolidinone Rings: Natural Products or Side-Reaction Products from Extraction Solvents?

**Mercedes Pérez-Bonilla [1,*], Marina Sánchez-Hidalgo [1], Ignacio González [1], Daniel Oves-Costales [1], Jesús Martín [1], José Murillo-Alba [1], José R. Tormo [1], Ahreum Cho [2], Soo-Young Byun [2], Joo-Hwan No [2], David Shum [2],
Jean-Robert Ioset [3], Olga Genilloud [1] and Fernando Reyes [1,*]**

[1] Fundación MEDINA, Avda. del Conocimiento 34, 18016 Granada, Spain; marina.sanchez@medinaandalucia.es (M.S.-H.); ignacio.gonzalez@medinaandalucia.es (I.G.);
daniel.oves@medinaandalucia.es (D.O.-C.); jesus.martin@medinaandalucia.es (J.M.); jose.murillo@medinaandalucia.es (J.M.-A.); ruben.tormo@medinaandalucia.es (J.R.T.); olga.genilloud@medinaandalucia.es (O.G.)

[2] Institut Pasteur Korea, 16, Daewangpangyo-ro 712 beon-gil, Bundang-gu, Seongnam-si 13488, Gyeonggi-do, Republic of Korea; ahreum.cho@ip-korea.org (A.C.); sooyoung.byun@ip-korea.org (S.-Y.B.); joohwan.no@ip-korea.org (J.-H.N.); david.shum@ip-korea.org (D.S.)

[3] Drugs for Neglected Diseases Initiative, 15 Chemin Camille-Vidart, 1202 Geneva, Switzerland;
jrioset@dndi.org

* Correspondence: mercedes.perez@medinaandalucia.es (M.P.-B.); fernando.reyes@medinaandalucia.es (F.R.); Tel.: +34-958993965 (ext. 7006) (F.R.)

Supporting Information Table of Contents

Contents Page

Figure S1. Chromatographic profiles at 210 nm of the D-FDVA adducts of serine references for the compound **2**……………………………………………………………………………………………………3
Figure S2. Chromatographic profiles at 210 nm of the single D-FDVA adducts of ornithine references for the compound **2**……………………..…………………………………………………..…………………4
Figure S3. Chromatographic profiles at 210 nm of the D-FDVA adducts for the compound **2**………………………………………………………………......……………………………………..5
Figure S4. Chromatographic profiles at 210 nm of the L-FDVA adducts for the compound **2**………………………………………………………………………………………………..…………..5
Figure S5. Chromatographic profiles at 210 nm of the D-FDVA adducts of serine references for the compounds **1**, **3** and **4**……………………………………………………………………………...…………6
Figure S6. Chromatographic profiles at 210 nm of the single D-FDVA adducts of ornithine references for the compounds **1**, **3** and **4**………………………………………………………………………………..……6
Figure S7. Chromatographic profiles at 210 nm of the D-FDVA adducts for the compound **1** …..……..7
Figure S8. Ion extraction of serine-D-FDVA adducts in compound **1**………………………………………7
Figure S9. Ion extraction of the double ornithine-D-FDVA adducts in compound **1**…..………...……….8
Figure S10. Ion extraction of the double *N*α-methyl-ornithine-D-FDVA adducts in compound **1**………9
Figure S11. Chromatographic profiles at 210 nm of the D-FDVA adducts for the compound **3**……...…10
Figure S12. Ion extraction of serine-D-FDVA adducts in compound **3**………………………………….....10
Figure S13. Ion extraction of the double ornithine-D-FDVA adducts in compound **3**…………………..…11
Figure S14. Ion extraction of the double *N*α-methyl-ornithine-D-FDVA adducts in compound **3**…..…..11
Figure S15. Chromatographic profiles at 210 nm of the D-FDVA adducts for the compound **4**…..…….12
Figure S16. Ion extraction of serine-D-FDVA adducts in compound **4**……………………………………12
Figure S17. Ion extraction of the double ornithine-D-FDVA adducts in compound **4**…………………...13
Figure S18. Ion extraction of the double *N*α-methyl-ornithine-D-FDVA adducts in compound **4**…..…13
Table S1. ORFs present in the madurastatin BGC from *Actinomadura* sp.CA-135719…………......…14-15
Figure S19. Comparison of the NRPS genes from *mad, rene*, and *mds* clusters…………………………....16
Figure S20. Scheme of side reaction involved in dimethyl-labeling of peptides with formaldehyde…..16
Figure S21. Scheme of the formation reaction to produce madurastatins H2 (**2**) and D2 (**5**)……………..17
Figure S22. Scheme of the formation reaction to produce 33-epimadurastatin D1 (**3**) and madurastatin D1 (**4**)……………………………………………………………………………………..…………..…..17
Figure S23. ^{1}H-NMR (500 MHz, dimethyl sulfoxide-d_6) spectrum of madurastatin H2 (**2**)……….……18
Figure S24. ^{13}C-NMR (125 MHz, dimethyl sulfoxide-d_6) spectrum of madurastatin H2 (**2**)…………….19
Figure S25. HSQC (dimethyl sulfoxide-d_6) spectrum of madurastatin H2 (**2**)………………….………..20
Figure S26. HMBC (dimethyl sulfoxide-d_6) spectrum of madurastatin H2 (**2**)……………..……………..21
Figure S27. COSY (dimethyl sulfoxide-d_6) spectrum of madurastatin H2 (**2**)………………..……………22
Figure S28. TOCSY (dimethyl sulfoxide-d_6) spectrum of madurastatin H2 (**2**)………………..…………23
Figure S29. ^{1}H-NMR (500 MHz, dimethyl sulfoxide-d_6) spectrum of 33 epi-madurastatin D1 (**3**)….….24
Figure S30. HSQC (dimethyl sulfoxide-d_6) spectrum of 33 epi-madurastatin D1 (**3**)……………..………25
Figure S31. ^{1}H-NMR (500 MHz, dimethyl sulfoxide-d_6) spectrum of madurastatin D1 (**4**)……….…….26
Figure S32. HSQC (dimethyl sulfoxide-d_6) spectrum of madurastatin D1 (**4**)……………………………27
Figure S33. HRMS spectrum of madurastatin H2 (**2**)……………………………………………………..28
Figure S34. HRMS spectrum of 33-*epi*-madurastatin D1 (**3**)……………………………………………...28

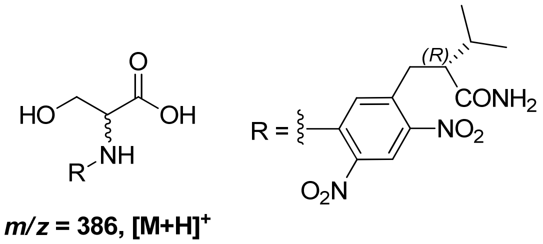

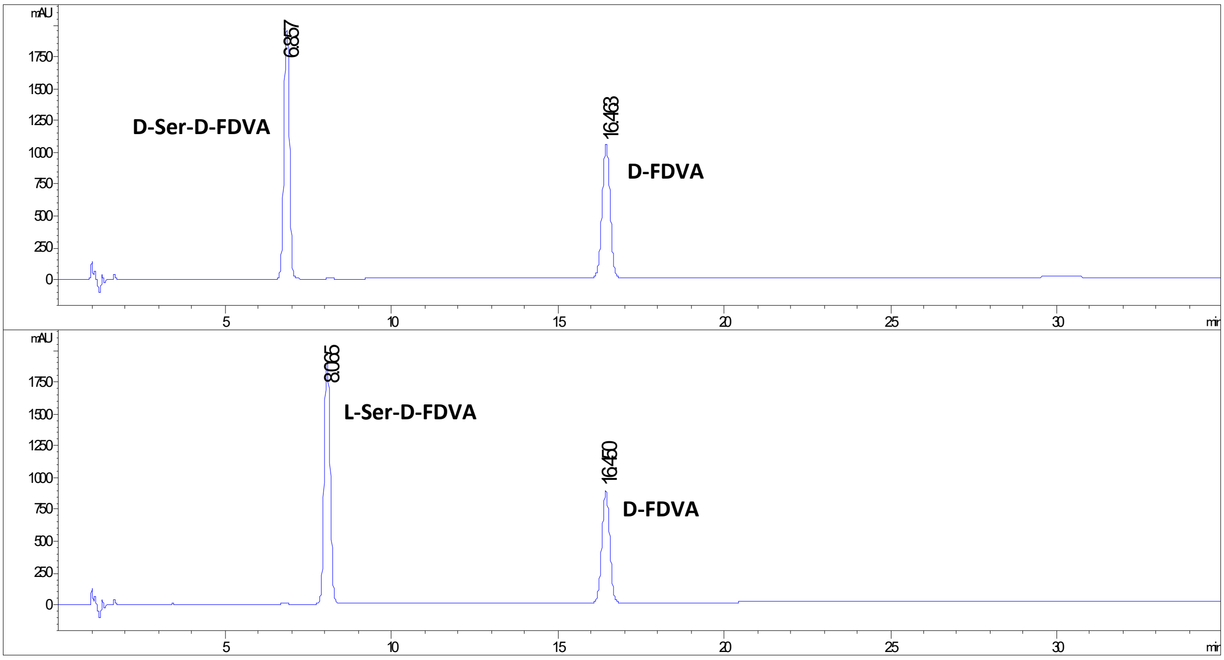

Figure S1. Chromatographic profiles at 210 nm of the D-FDVA adducts of serine references: $[M+H]^+$ = 386, $[M+Na]^+$ = 408; D-serine (RT = 6.857 min) and L-serine (RT = 8.065 min) for the compound **2**.

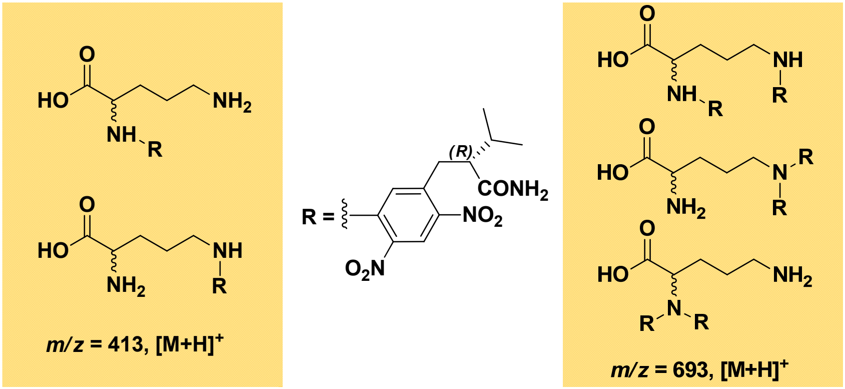

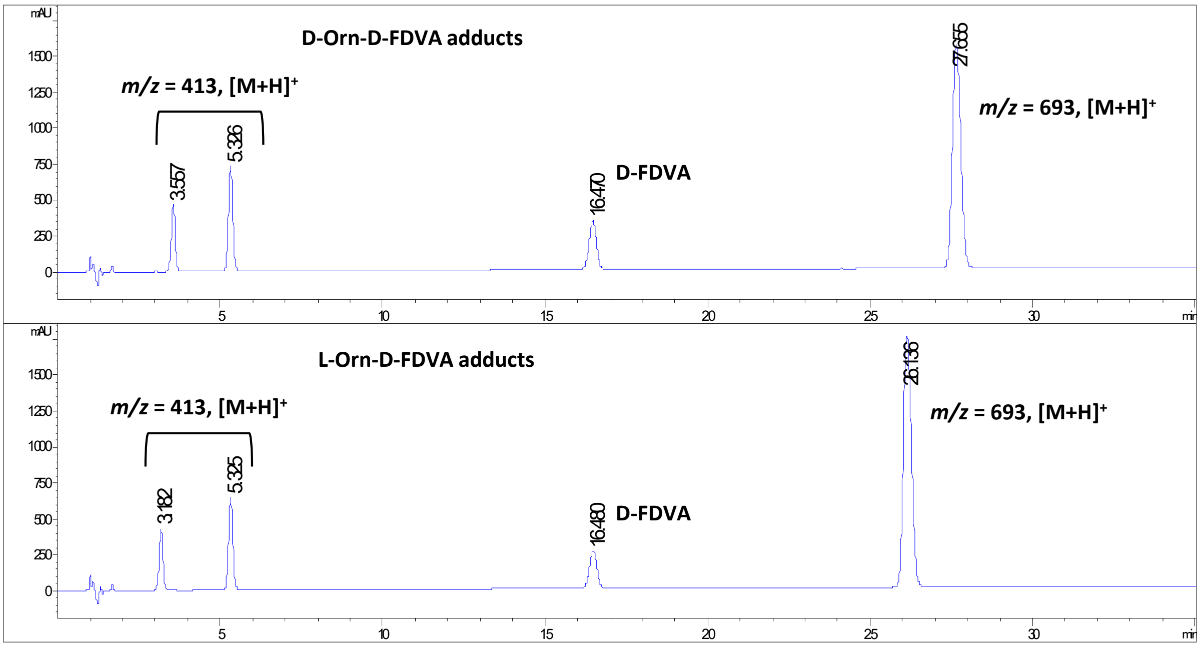

Figure S2. Chromatographic profiles at 210 nm of the single D-FDVA adducts of ornithine references: D-ornithine (RT = 3.557 and 5.326 min) and L-ornithine (RT = 3.182 and 5.325 min). Double D-FDVA adducts of ornithine references: D-ornithine (RT = 27.655 min) and L-ornithine (RT = 26.136 min) for the compound **2**.

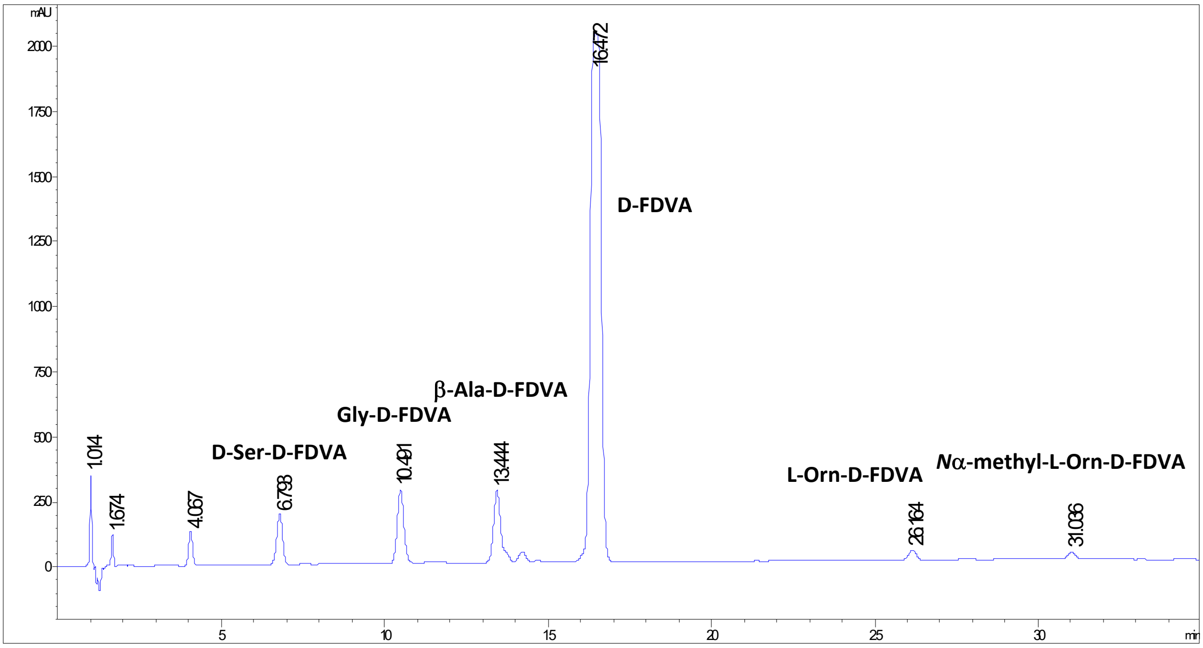

Figure S3. Chromatographic profiles at 210 nm of the D-FDVA adducts for the compound **2**: D-serine (RT = 6.793 min, *m/z* 408 [M+Na]⁺, *m/z* 386 [M+H]⁺), glycine (RT = 10.491 min, *m/z* 378 [M+Na]⁺, *m/z* 356 [M+H]⁺), β-alanine (RT = 13.444 min, *m/z* 392 [M+Na]⁺, *m/z* 370 [M+H]⁺), double D-FDVA adduct of L-ornithine (RT = 26.164 min, *m/z* 715 [M+Na]⁺, *m/z* 693 [M+H]⁺), double D-FDVA adduct of *N*α-methyl-L-ornithine (RT = 31.036 min, *m/z* 729 [M+Na]⁺, *m/z* 707 [M+H]⁺).

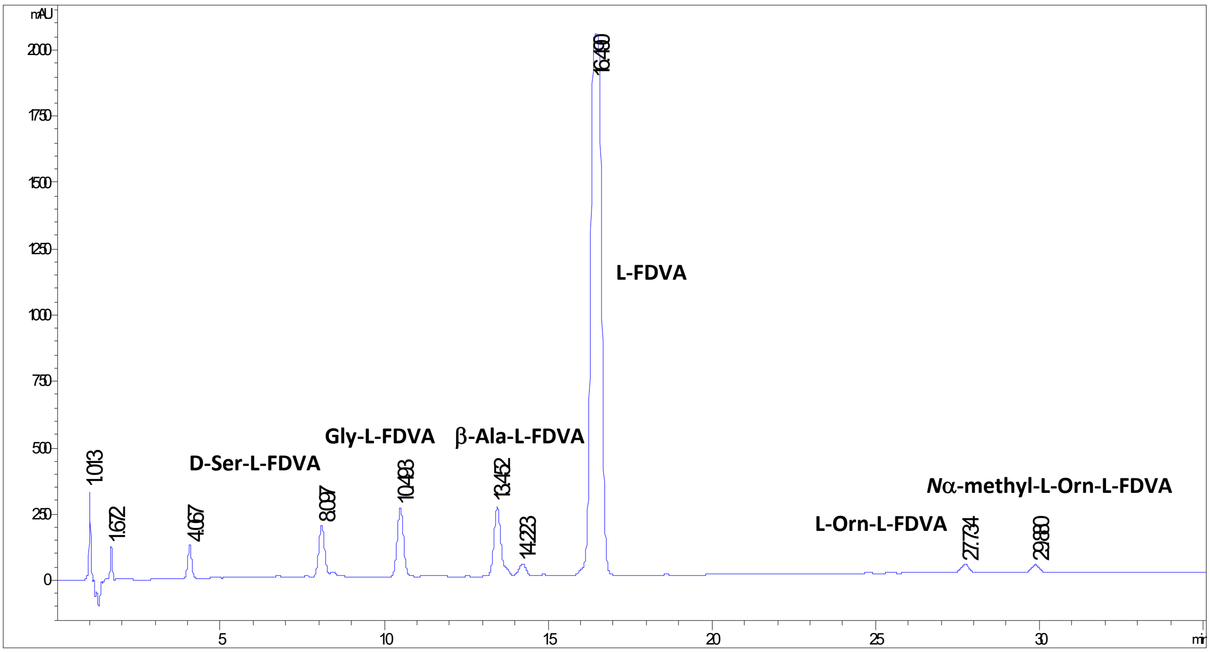

Figure S4. Chromatographic profiles at 210 nm of the L-FDVA adducts for the compound **2**: D-serine (RT = 8.097 min, *m/z* 408 [M+Na]⁺, *m/z* 386 [M+H]⁺), glycine (RT = 10.493 min, *m/z* 378 [M+Na]⁺, *m/z* 356 [M+H]⁺), β-alanine (RT = 13.452 min, *m/z* 392 [M+Na]⁺, *m/z* 370 [M+H]⁺), double L-FDVA adduct of L-ornithine (RT = 27.734 min, *m/z* 715 [M+Na]⁺, *m/z* 693 [M+H]⁺), double L-FDVA adduct of *N*α-methyl-L-ornithine (RT = 29.880 min, *m/z* 729 [M+Na]⁺, *m/z* 707 [M+H]⁺).

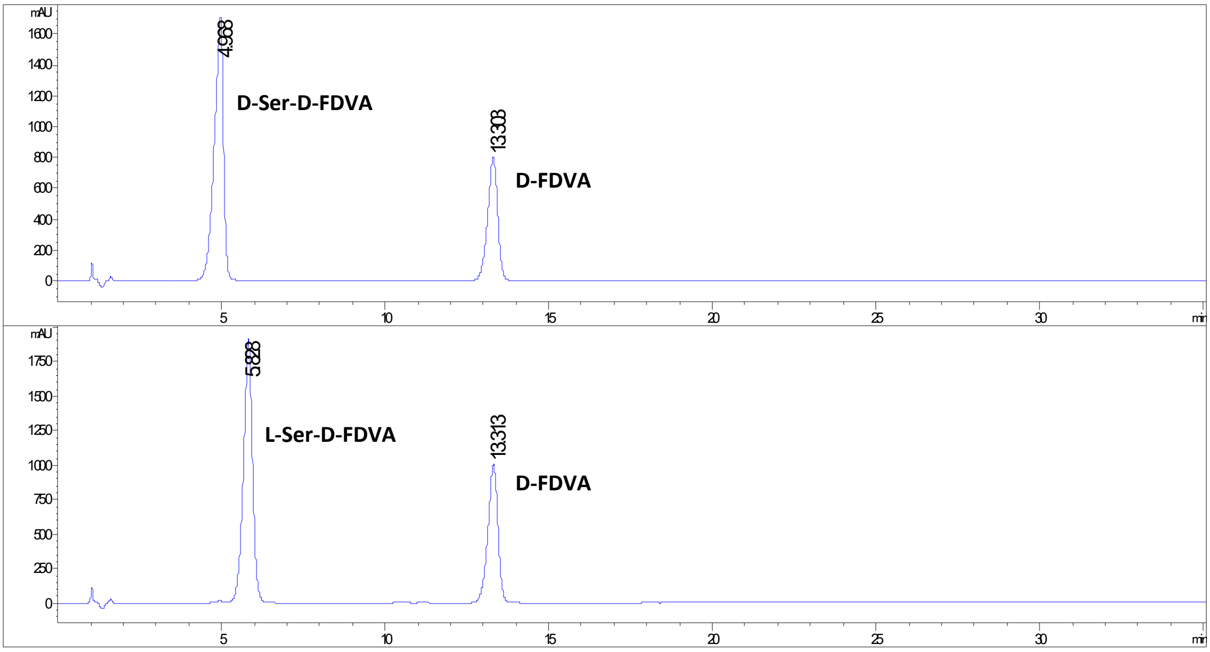

Figure S5. Chromatographic profiles at 210 nm of the D-FDVA adducts of serine references: $[M+H]^+$ = 386, $[M+Na]^+$ = 408; D-serine (RT = 4.968 min) and L-serine (RT = 5.828 min) for the compounds **1**, **3** and **4**.

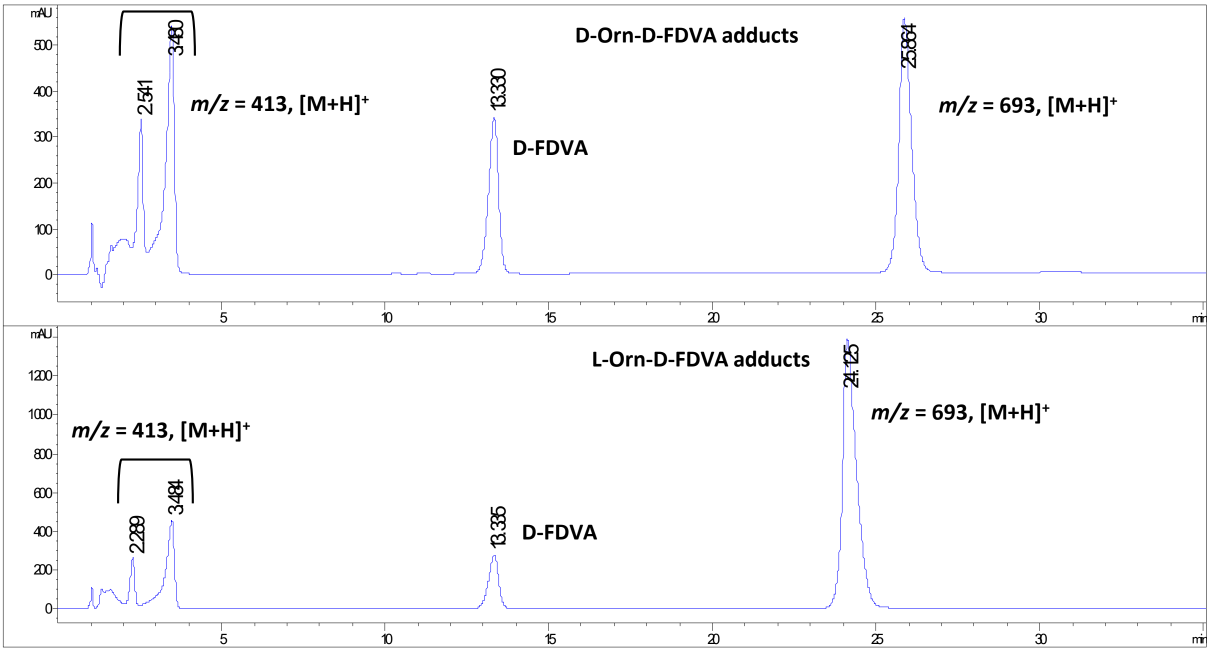

Figure S6. Chromatographic profiles at 210 nm of the single D-FDVA adducts of ornithine references: D-ornithine (RT = 2.541 and 3.480 min) and L-ornithine (RT = 2.289 and 3.484 min). Double D-FDVA adducts of ornithine references: D-ornithine (RT = 25.864 min) and L-ornithine (RT = 24.125 min) for the compounds **1**, **3** and **4**.

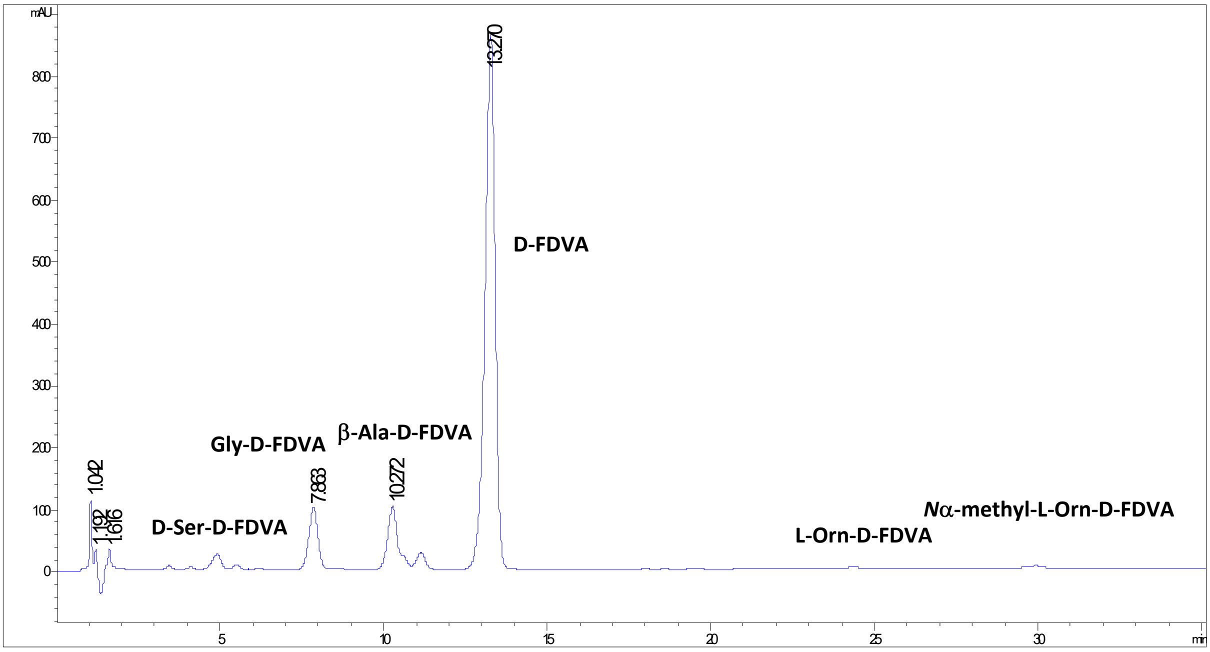

Figure S7. Chromatographic profiles at 210 nm of the D-FDVA adducts for the compound **1**: D-serine (RT = 4.940 min, *m/z* 408 $[M+Na]^+$, *m/z* 386 $[M+H]^+$), glycine (RT = 7.863 min, *m/z* 378 $[M+Na]^+$, *m/z* 356 $[M+H]^+$), β-alanine (RT = 10.272 min, *m/z* 392 $[M+Na]^+$, *m/z* 370 $[M+H]^+$), double D-FDVA adduct of L-ornithine (RT = 24.425 min, *m/z* 715 $[M+Na]^+$, *m/z* 693 $[M+H]^+$), double D-FDVA adduct of *N*α-methyl-L-ornithine (RT = 29.972 min, *m/z* 729 $[M+Na]^+$, *m/z* 707 $[M+H]^+$).

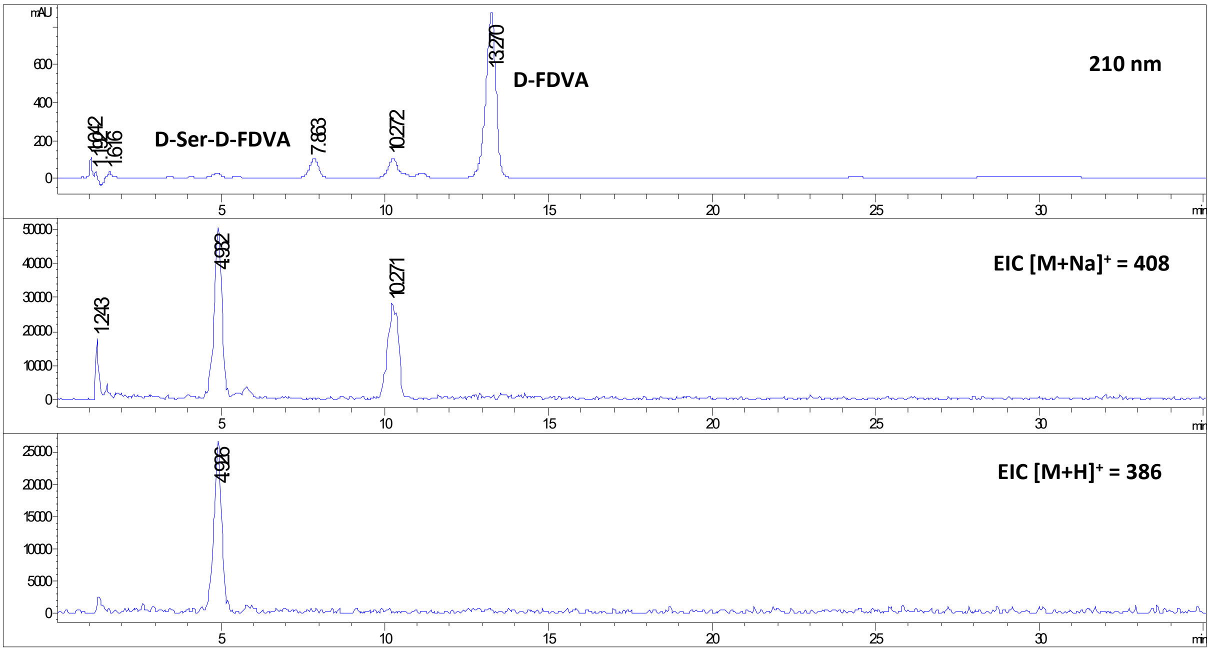

Figure S8. Ion extraction of serine-D-FDVA adducts in compound **1**: D-serine (RT = 4.932 min, *m/z* 408 $[M+Na]^+$, *m/z* 386 $[M+H]^+$).

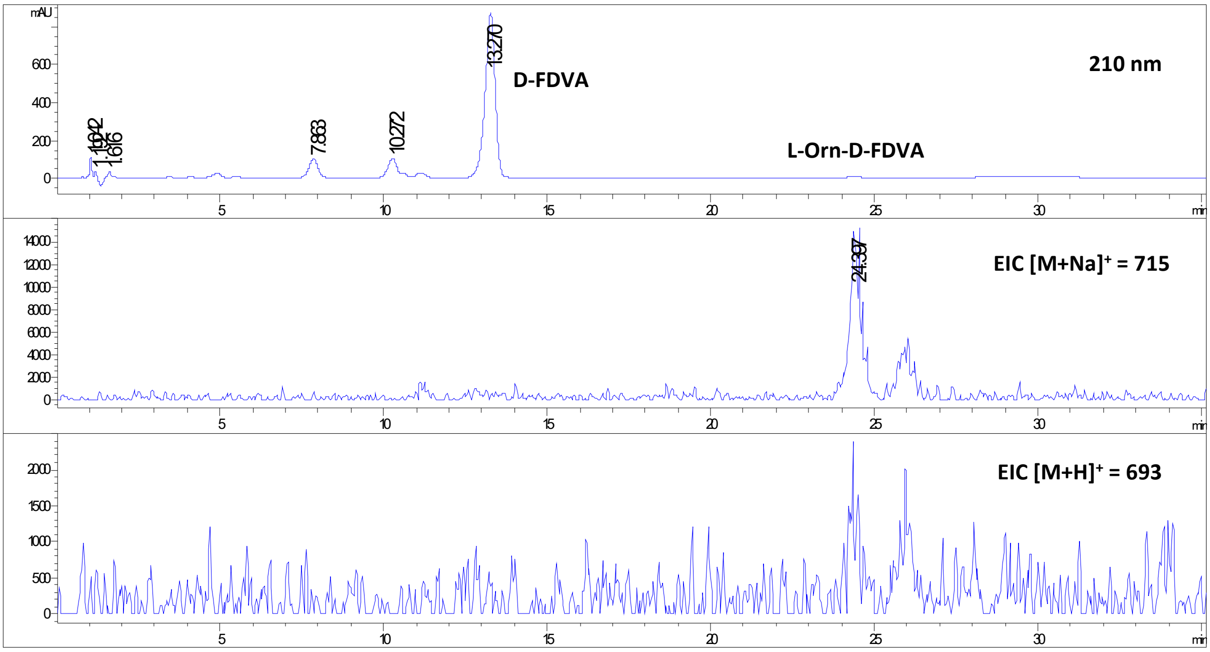

Figure S9. Ion extraction of the double ornithine-D-FDVA adducts in compound **1**: L-ornithine (RT = 24.397 min, *m*/*z* 715 [M+Na]+, *m*/*z* 693 [M+H]+).

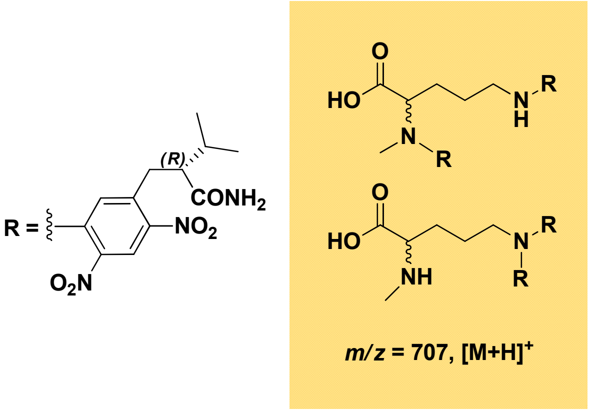

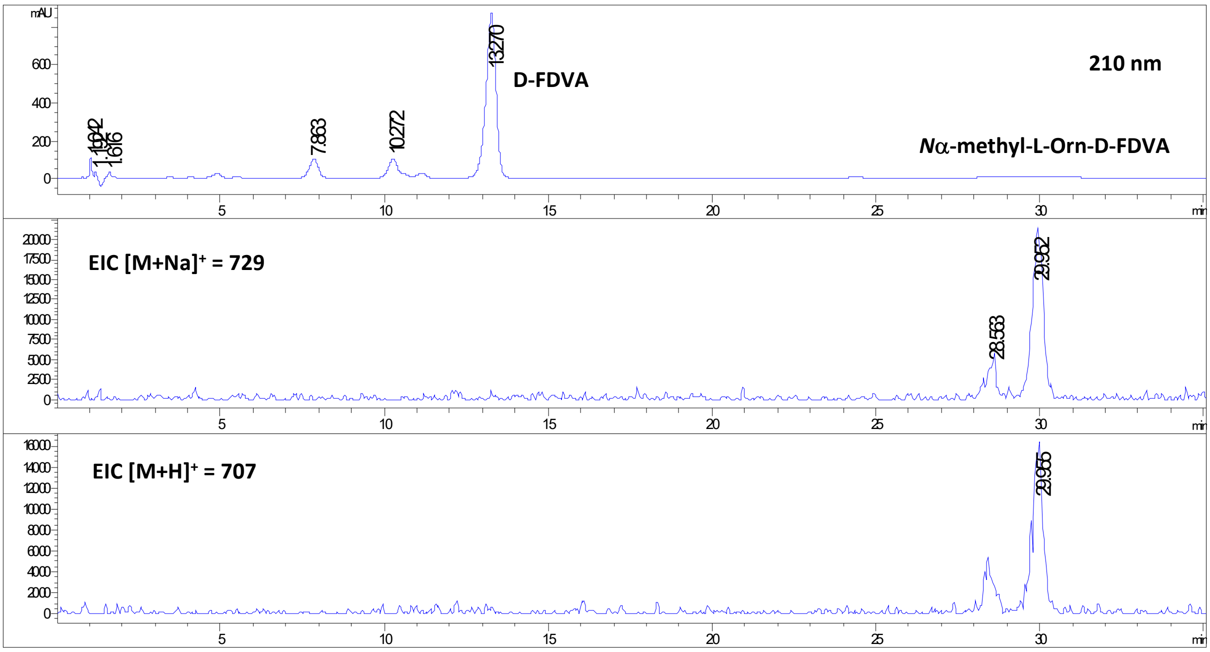

Figure S10. Ion extraction of the double *N*α-methyl-ornithine-D-FDVA adducts in compound **1**: *N*α-methyl-L-ornithine (RT = 29.952 min, *m/z* 729 [M+Na]+, *m/z* 707 [M+H]+).

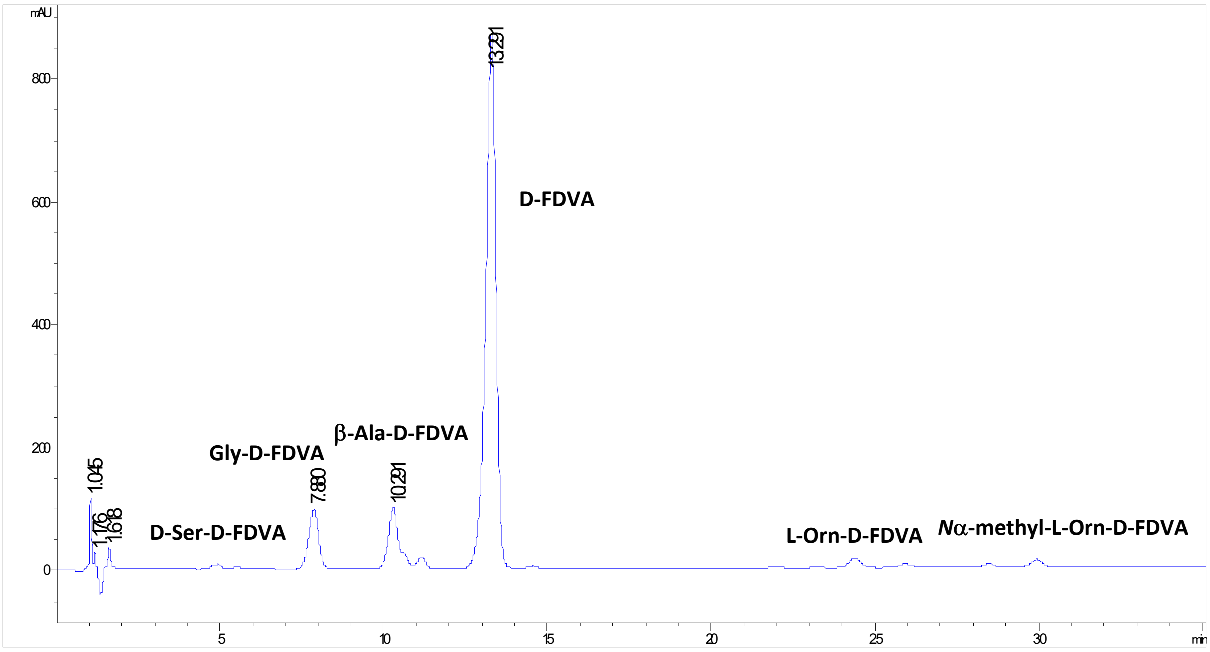

Figure S11. Chromatographic profiles at 210 nm of the D-FDVA adducts for the compound **3**: D-serine (RT = 4.931 min, *m*/*z* 408 $[M+Na]^+$, *m*/*z* 386 $[M+H]^+$), glycine (RT = 7.880 min, *m*/*z* 378 $[M+Na]^+$, *m*/*z* 356 $[M+H]^+$), β-alanine (RT = 10.291 min, *m*/*z* 392 $[M+Na]^+$, *m*/*z* 370 $[M+H]^+$), double D-FDVA adduct of L-ornithine (RT = 24.405 min, *m*/*z* 715 $[M+Na]^+$, *m*/*z* 693 $[M+H]^+$), double D-FDVA adduct of *N*α-methyl-L-ornithine (RT = 29.984 min, *m*/*z* 729 $[M+Na]^+$, *m*/*z* 707 $[M+H]^+$).

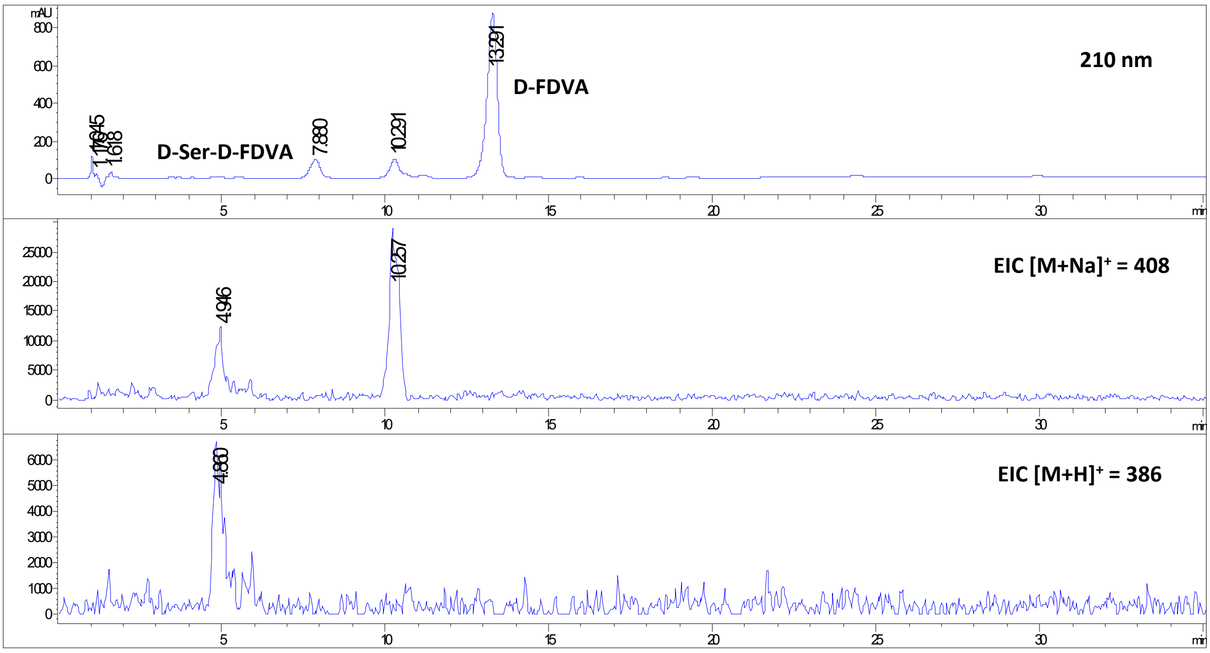

Figure S12. Ion extraction of serine-D-FDVA adducts in compound **3**: D-serine (RT = 4.931 min, *m*/*z* 408 $[M+Na]^+$, *m*/*z* 386 $[M+H]^+$).

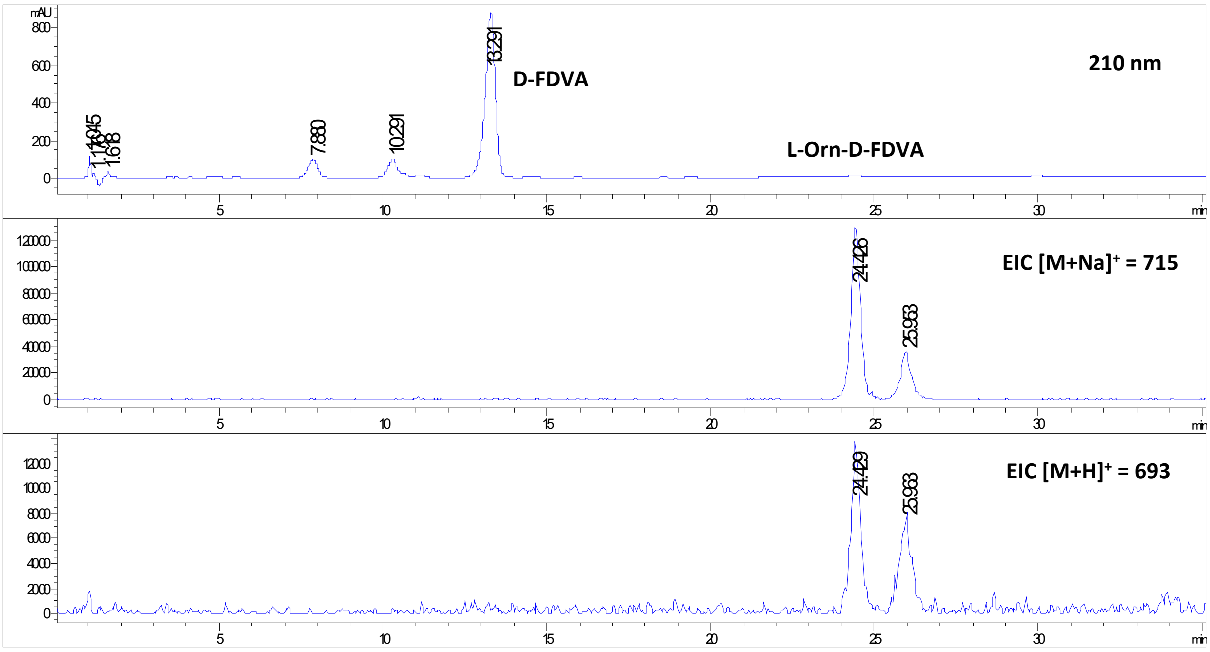

Figure S13. Ion extraction of the double ornithine-D-FDVA adducts in compound **3**: L-ornithine (RT = 24.426 min, *m*/*z* 715 [M+Na]+, *m*/*z* 693 [M+H]+).

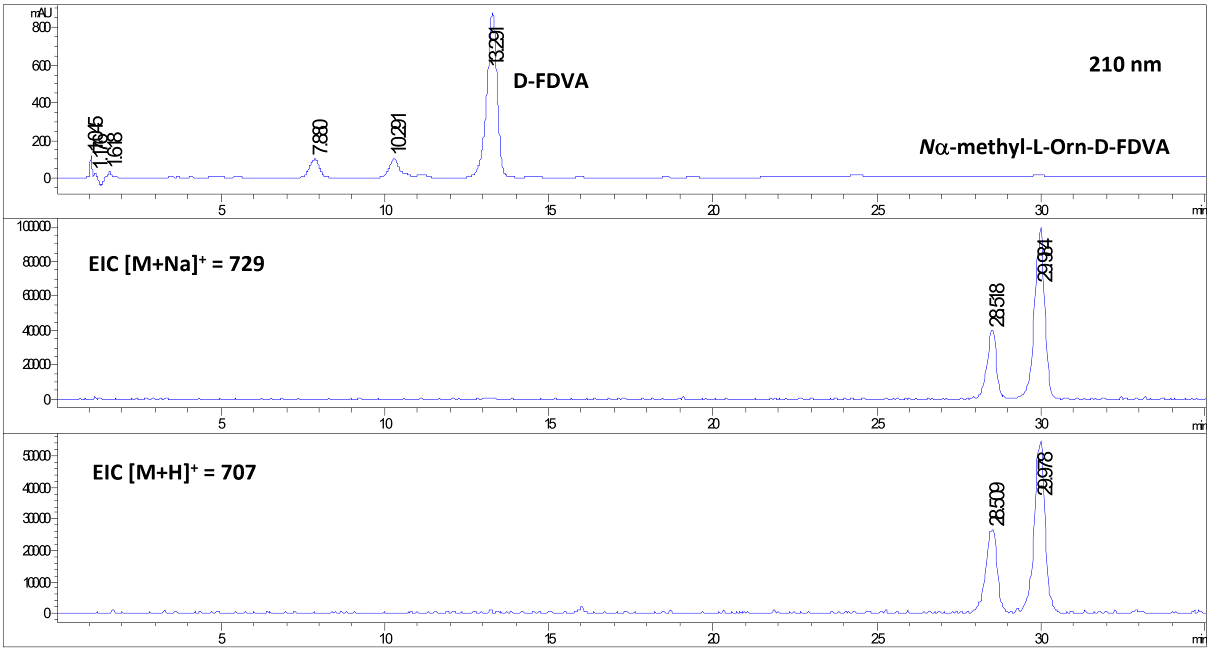

Figure S14. Ion extraction of the double *N*α-methyl-ornithine-D-FDVA adducts in compound **3**: *N*α-methyl-L-ornithine (RT = 29.978 min, *m*/*z* 729 [M+Na]+, *m*/*z* 707 [M+H]+).

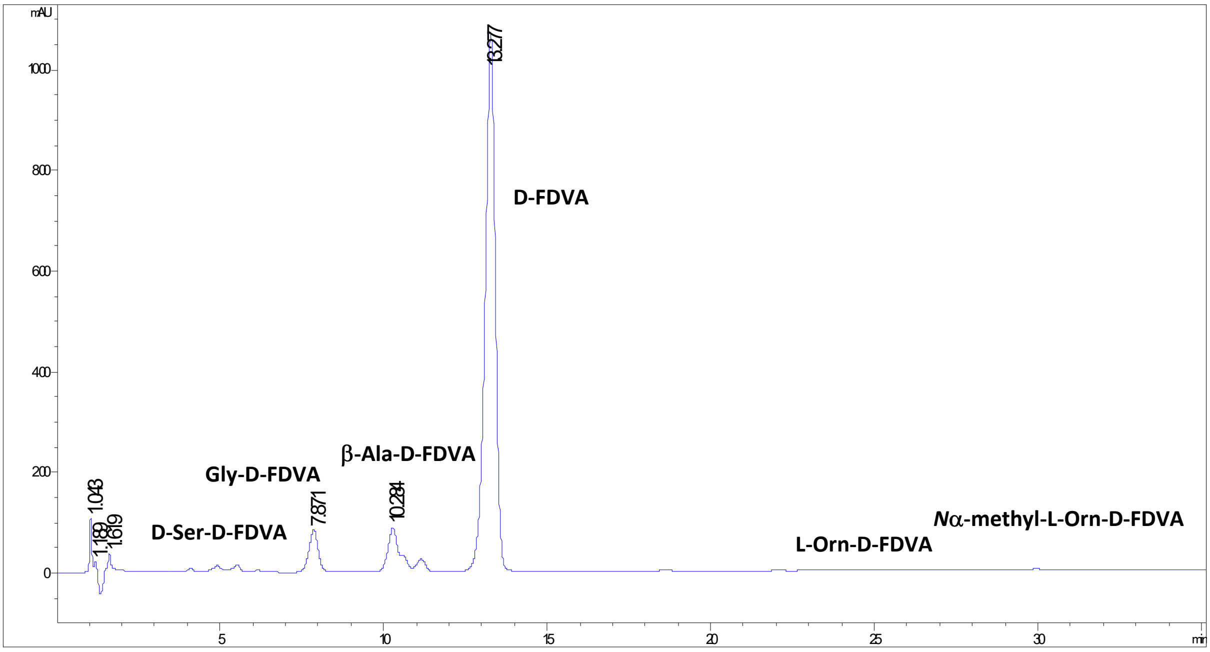

Figure S15. Chromatographic profiles at 210 nm of the D-FDVA adducts for the compound **4**: D-serine (RT = 4.911 min, *m*/*z* 408 [M+Na]+, *m*/*z* 386 [M+H]+), glycine (RT = 7.871 min, *m*/*z* 378 [M+Na]+, *m*/*z* 356 [M+H]+), β-alanine (RT = 10.272 min, *m*/*z* 392 [M+Na]+, *m*/*z* 370 [M+H]+), double D-FDVA adduct of L-ornithine (RT = 24.469 min, *m*/*z* 715 [M+Na]+, *m*/*z* 693 [M+H]+), double D-FDVA adduct of *N*α-methyl-L-ornithine (RT = 29.857 min, *m*/*z* 729 [M+Na]+, *m*/*z* 707 [M+H]+).

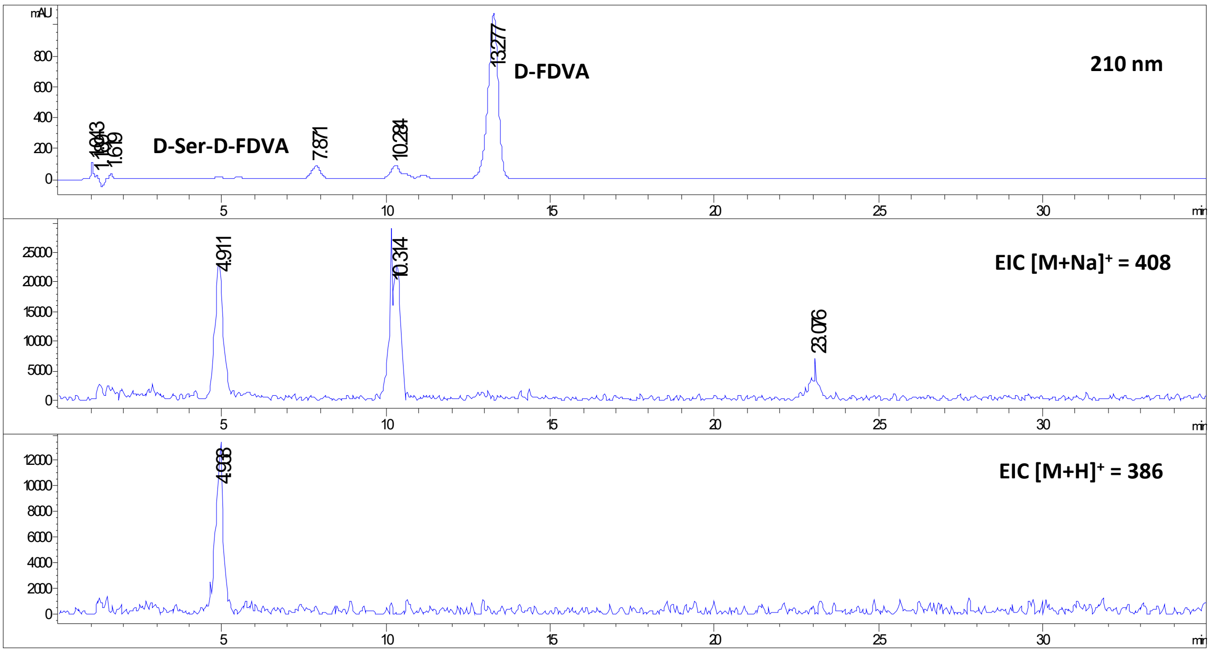

Figure S16. Ion extraction of serine-D-FDVA adducts in compound **4**: D-serine (RT = 4.911 min, *m*/*z* 408 [M+Na]+, *m*/*z* 386 [M+H]+).

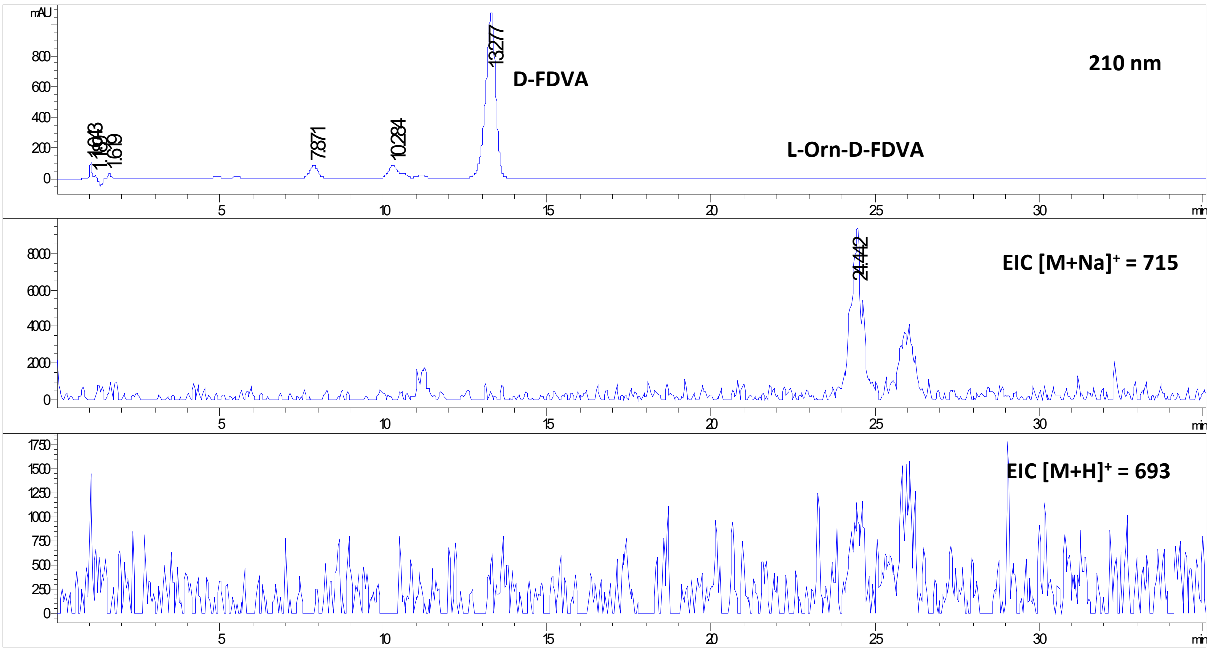

Figure S17. Ion extraction of the double ornithine-D-FDVA adducts in compound **4**: L-ornithine (RT = 24.442 min, *m/z* 715 [M+Na]+, *m/z* 693 [M+H]+).

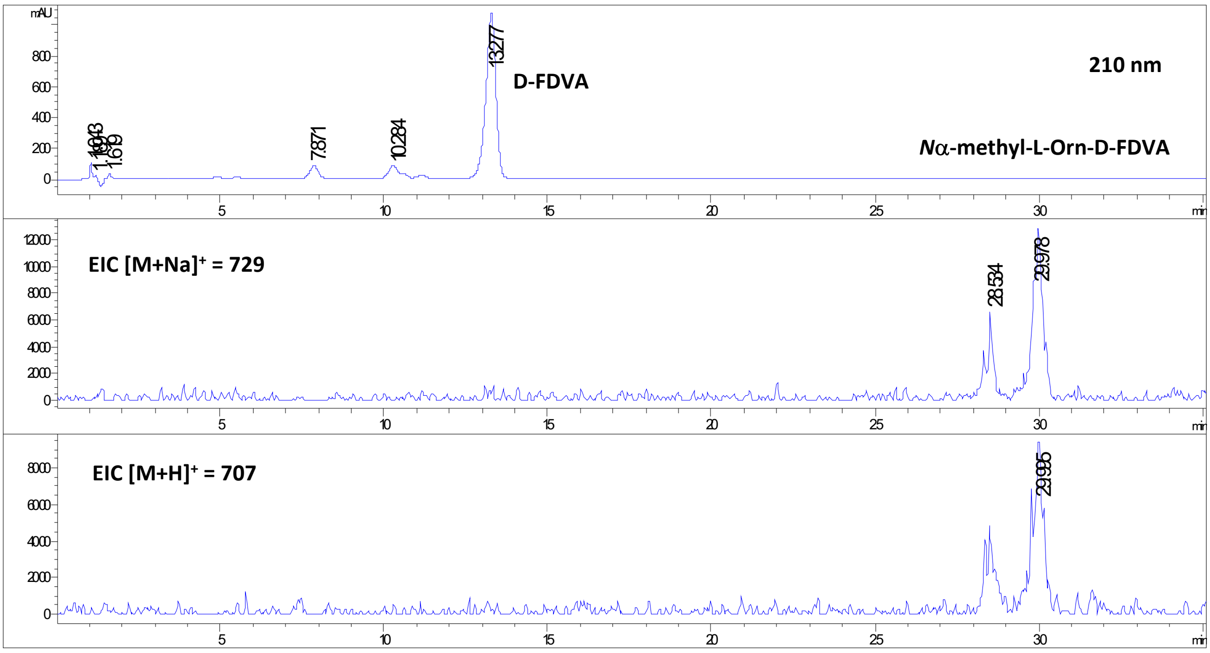

Figure S18. Ion extraction of the double *N*α-methyl-ornithine-D-FDVA adducts in compound **4**: *N*α-methyl-L-ornithine (RT = 29.978 min, *m/z* 729 [M+Na]+, *m/z* 707 [M+H]+).

Table S1. ORFs present in the madurastatin BGC from *Actinomadura* sp.CA-135719. A comparison with the ORFs present in the *mad* and *rene* BGCs is shown.

mds gene	Function	Length (bp)	Closest BLAST homolog			Homologies with *mad* cluster (% identity/ similarity)	Homologies with *red* cluster (% identity/ similarity)
			Reference	Strain	%Identity/Similarity		
1	Maf family protein	600	WP_163063977.1	*Actinomadura bangladeshensis*	84/88		
2	Inositol monophosphatase	834	WP_163063976.1	*Actinomadura bangladeshensis*	86/89		
3	Predicted NTPase (NACHT family)	2289	CNG46166.1	*Mycobacterium tuberculosis*	83/88		
4	TetR/AcrR family transcriptional regulator	633	WP_250355147.1	*Actinomadura madurae*	95/96		
5	STAS domain-containing protein	396	WP_021597847.1	*Actinomadura madurae*	87/90	Mad69 (77/84)	
6	PaaX family transcriptional regulator C-terminal domain-containing protein	852	WP_200871800.1	*Actinomadura madurae*	97/98		RB99_01649 (82/87)
7	ABC transporter substrate-binding protein	1197	WP_132199655.1	*Actinomadura darangshiensis*	93/97		
8	ABC transporter ATP-binding protein	768	WP_132199656.1	*Actinomadura darangshiensis*	95/97		
9	ABC transporter ATP-binding protein	777	WP_111831484.1	*Actinomadura madurae*	88/90		
10	branched-chain amino acid ABC transporter permease	864	WP_250370415.1	*Actinomadura madurae*	96/98		
11	branched-chain amino acid ABC transporter permease	1047	WP_176405448.1	*Actinomadura* sp. BRA 177	90/93		
12	long-chain fatty acid--CoA ligase	1503	WP_250355151.1	*Actinomadura madurae*	93/96		
13	IclR family transcriptional regulator	795	WP_111831486.1	*Actinomadura madurae*	93/93		RB99_01648 (83/89)
14	IclR family transcriptional regulator C-terminal domain-containing protein	1020	WP_250355152.1	*Actinomadura madurae*	94/98		RB99_01647 (87/91)
15	hypothetical protein	147	WP_021597837.1	*Actinomadura madurae*	90/95		RB99_01646 (81/89)
16	ABC transporter permease	1638	WP_250370418.1	*Actinomadura madurae*	92/95	Mad68 (77/85)	RB99_01645 (78/86)
17	ABC transporter ATP-binding protein	903	WP_021599067.1	*Actinomadura madurae*	95/97	Mad67 (90/94)	RB99_01644 (89/93)
18	TetR/AcrR family transcriptional regulator	675	WP_021599066.1	*Actinomadura madurae*	89/93	Mad66 (78/85)	RB99_01643 (75/82)
19	iron chelate uptake ABC transporter family permease subunit	1050	NVI90222.1	*Actinomadura* sp. BRA 177	85/89	Mad65 (86/89)	RB99_01642 (80/86)
20	Iron chelate uptake ABC transporter family permease subunit	1020	WP_132160734.1	*Actinomadura* sp. 7K507	88/93	Mad64 (90/94)	RB99_01641 (86/90)
21	non-ribosomal peptide synthetase	3366	WP_111831488.1	*Actinomadura madurae*	92/93	Mad63 (87/91)	ReneQ (83/87)
22	Enterobactin transporter EntS	1248	WP_111831489.1	*Actinomadura madurae*	95/97	Mad62 (88/93)	ReneP (79/83)
23	aspartate 1-decarboxylase	387	WP_250370422.1	*Actinomadura madurae*	98/99	Mad61 (94/98)	ReneO (85/91)
24	AMP-binding protein	1581	WP_021593700.1	*Actinomadura madurae*	95/97	Mad60 (87/92)	ReneN (82/90)
25	NBR1-Ig-like domain-containing protein	906	WP_250355158.1	*Actinomadura madurae*	79/83		
26	Enoyl-CoA hydratase/isomerase family protein	414	WP_075021693.1	*Actinomadura madurae*	78/86		
27	glyoxalase	612	WP_075021694.1	*Actinomadura madurae*	88/93		
28	Unknown protein	411					
29	DUF418 domain-containing protein	975	NVI87458.1	*Actinomadura madurae*	83/87		
30	Unknown protein	564					
31	threonine/serine dehydratase	930	WP_088950310.1	*Micromonospora zamorensis*	64/74		
32	Hypothetical proline hydroxylase	816	WP_204927031.1	*Micromonospora humida*	49/63		
33	Acetyl transferase	468	KKP60044.1	*Candidatus Roizmanbacteria* bacterium GW2011_GWA2_34_18	55/71		
34	5'-methylthioadenosine/adenosylhomocysteine nucleosidase	771	WP_122919150.1	*Brevibacillus fluminis*	38/53		
35	Caspase family protein	4251	WP_040693739.1	*Nocardia vinacea*	42/54		
36	Salicylate synthase	1311	WP_021593683.1	*Actinomadura madurae*	87/92	Mad31 (82/88)	ReneM (76/83)

37	NRPS	11898	WP_024934912.1	*Actinomadura madurae*	92/94	Mad30 (86/89)	ReneL (80/85)
38	iron-siderophore ABC transporter substrate-binding protein	1026	WP_033331134.1	*Actinomadura madurae*	91/94	Mad29 (80/87)	ReneK (77/87)
39	lysine N(6)-hydroxylase/L-ornithine N(5)-oxygenase	1272	WP_250355177.1	*Actinomadura madurae*	95/97	Mad28 (89/92)	ReneJ (86/89)
40	MbtH family protein	198	WP_255273069.1	*Actinomadura madurae*	97/98	Mad27 (88/90)	ReneI (78/84)
41	PHB depolymerase family esterase	864	WP_021593677.1	*Actinomadura madurae*	89/93	Mad26 (81/90)	ReneH (80/90)
42	MFS transporter	1272	WP_151597199.1	*Actinomadura madurae*	90/95	Mad25 (90/91)	ReneG (90/95)
43	MarR family transcriptional regulator	474	WP_208269029.1	*Actinomadura nitritigenes*	82/88		ReneF (82/88)
44	Unknown protein	135					
45	phosphotransferase family protein	1032	WP_244938629.1	*Actinomadura madurae*	90/94		ReneC (79/85)
46	Unknown protein	129					
47	MaoC family dehydratase	453	WP_021593675.1	*Actinomadura madurae*	98/98		ReneB (85/92)
48	long-chain fatty acid--CoA ligase	1647	WP_250355181.1	*Actinomadura madurae*	95/97	Mad24 (91/96)	ReneA (90/94)
49	TetR/AcR family transcriptional regulator	609	WP_075021705.1	*Actinomadura madurae*	93/96	Mad23 (89/94)	
50	ABC transporter ATP-binding protein	780	WP_250355182.1	*Actinomadura madurae*	94/96	Mad22 (90/93)	
51	ABC transporter ATP-binding protein	708	WP_089328063.1	*Actinomadura madurae*	95/97	Mad21 (90/94)	
52	Branched-chain amino acid ABC transporter permease	879	WP_132203346.1	*Actinomadura darangshiensis*	97/98	Mad20 (95/96)	
53	Branched-chain amino acid ABC transporter permease	1206	WP_218163691.1	*Actinomadura darangshiensis*	97/97	Mad19 (88/91)	
54	ABC transporter substrate-binding protein	1278	WP_024934907.1	*Actinomadura madurae*	94/97	Mad18 (93/97)	
55	Transcriptional regulator, DeoR family	804	SFO45426.1	*Actinomadura madurae*	97/98	Mad17 (95/98)	
56	HPr family phosphocarrier protein	279	WP_021599327.1	*Actinomadura madurae*	98/98		
57	zinc-dependent dehydrogenase	1041	WP_021599328.1	*Actinomadura madurae*	98/99		
58	PTS system IIA component, Fru family	498	SFO45486.1	*Actinomadura madurae*	92/95		
59	PTS lactose transporter subunit IIB	300	WP_250355189.1	*Actinomadura madurae*	95/95		
60	PTS mannitol transporter subunit IICB	1242	WP_250355190.1	*Actinomadura madurae*	92/93		
61	fructose-1-phosphate kinase	939	SFO45542.1	*Actinomadura madurae*	98/98	Mad16 (98/97)	

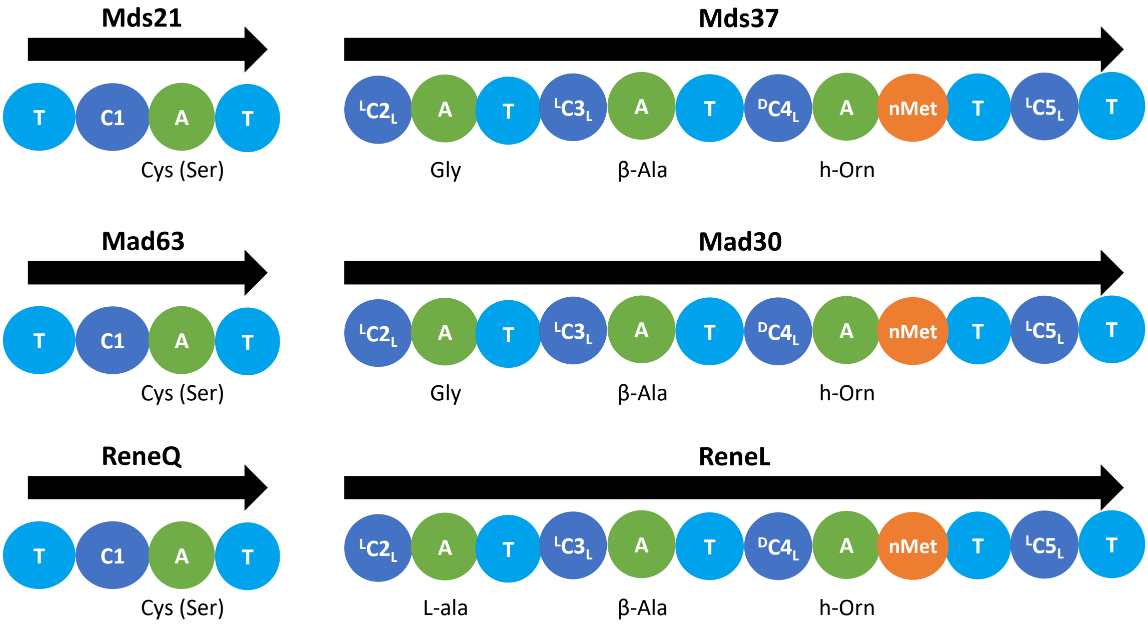

Figure S19. Comparison of the NRPS genes from *mad, rene,* and *mds* clusters. The predicted amino acids incorporated by each NRPS module are indicated.

The mechanism of 4-imidazolidinone formation in madurastatins is similar to that occurring when the *N*-terminal amine group of peptides reacts with formaldehyde to form a hydroxymethyl intermediate. This hydroxymethyl derivative undergoes dehydration to give an imine (Schiff-base), which is also the key intermediate for the demethylation reaction, and the production of the 4-imidazolidinone moiety (Figure S6).

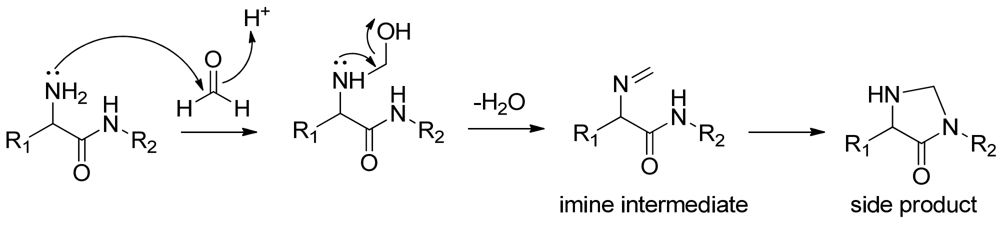

Figure S20. Scheme of side reaction involved in dimethyl-labeling of peptides with formaldehyde.

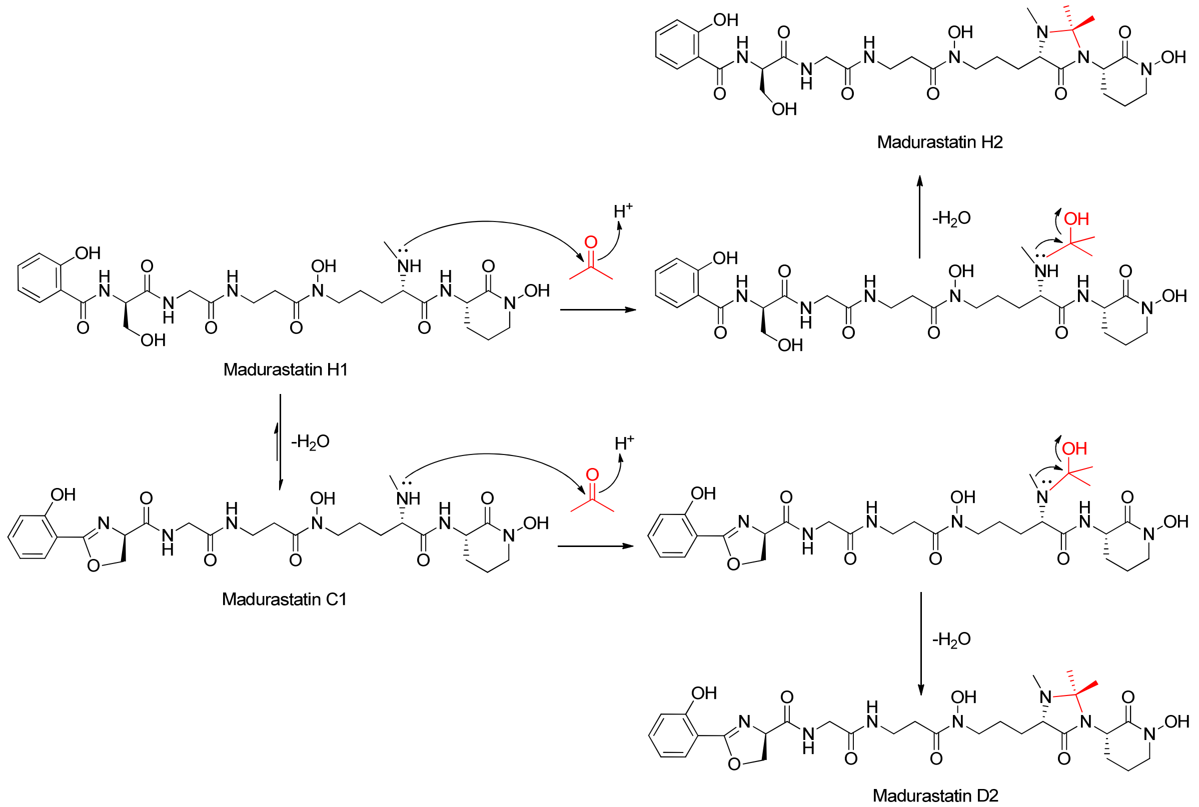

Figure S21. Scheme of the formation reaction to produce madurastatins H2 (**2**) and D2 (**5**).

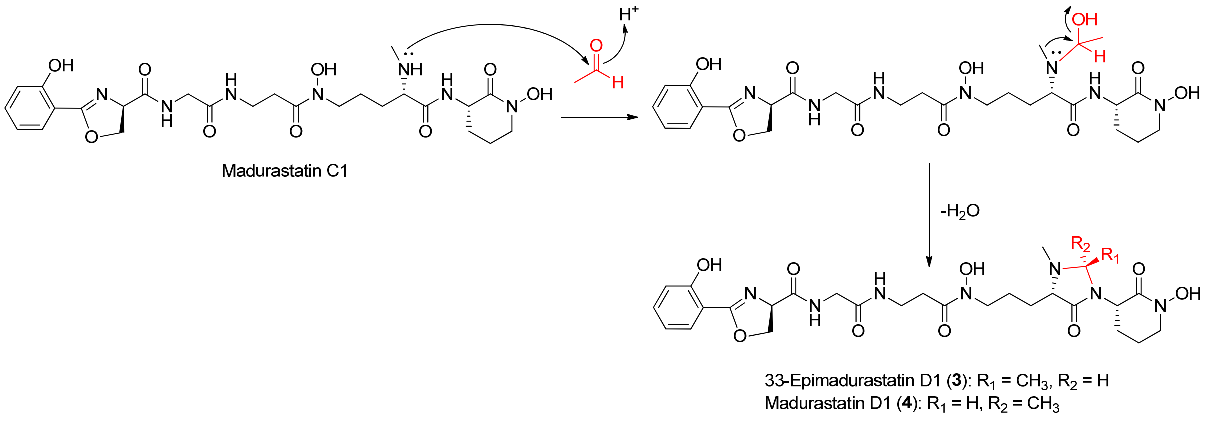

Figure S22. Scheme of the formation reaction to produce 33-epimadurastatin D1 (**3**) and madurastatin D1 (**4**).

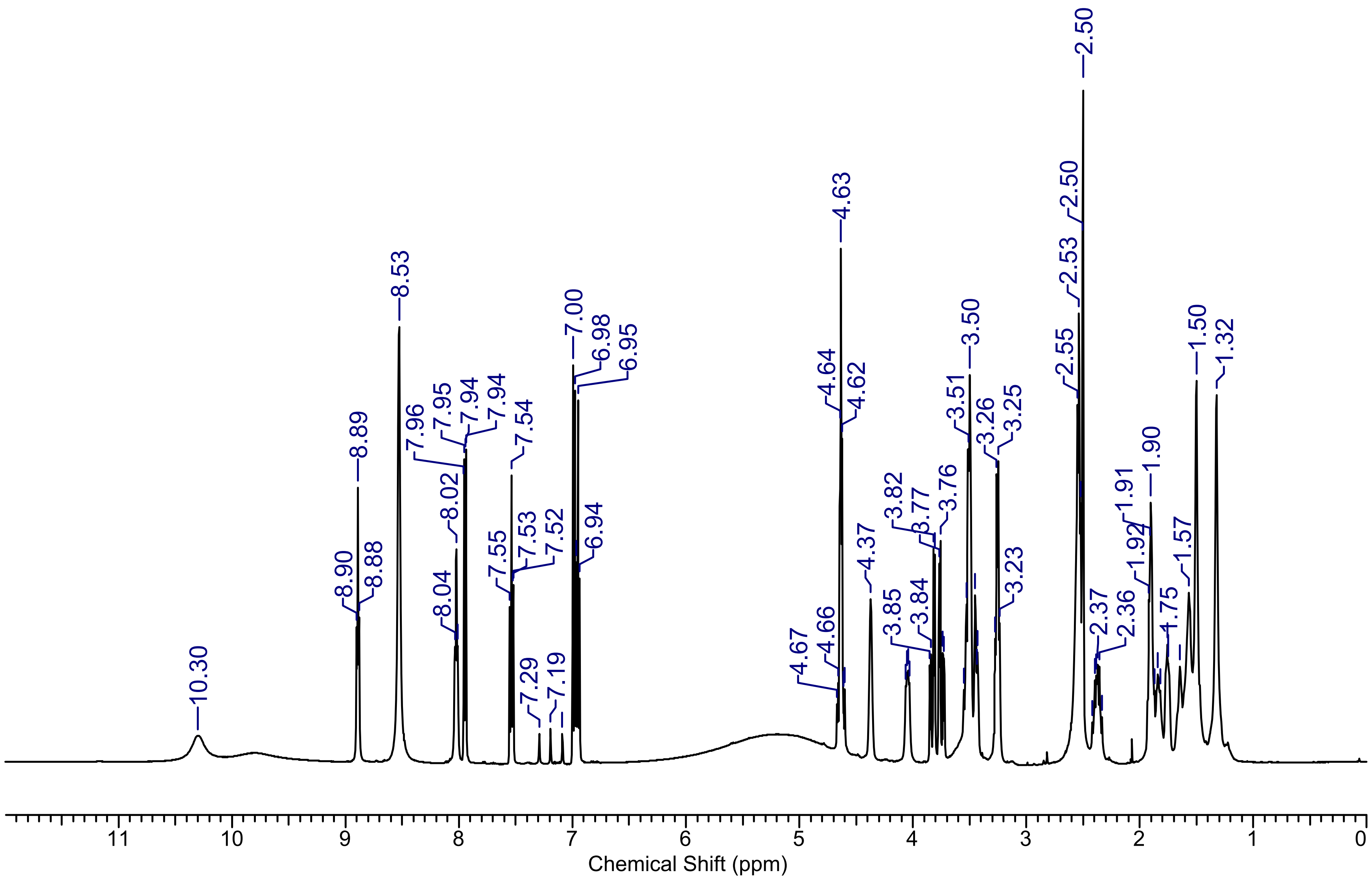

Figure S23. ^{1}H-NMR (500 MHz, dimethyl sulfoxide-d_6) spectrum of madurastatin H2 (**2**).

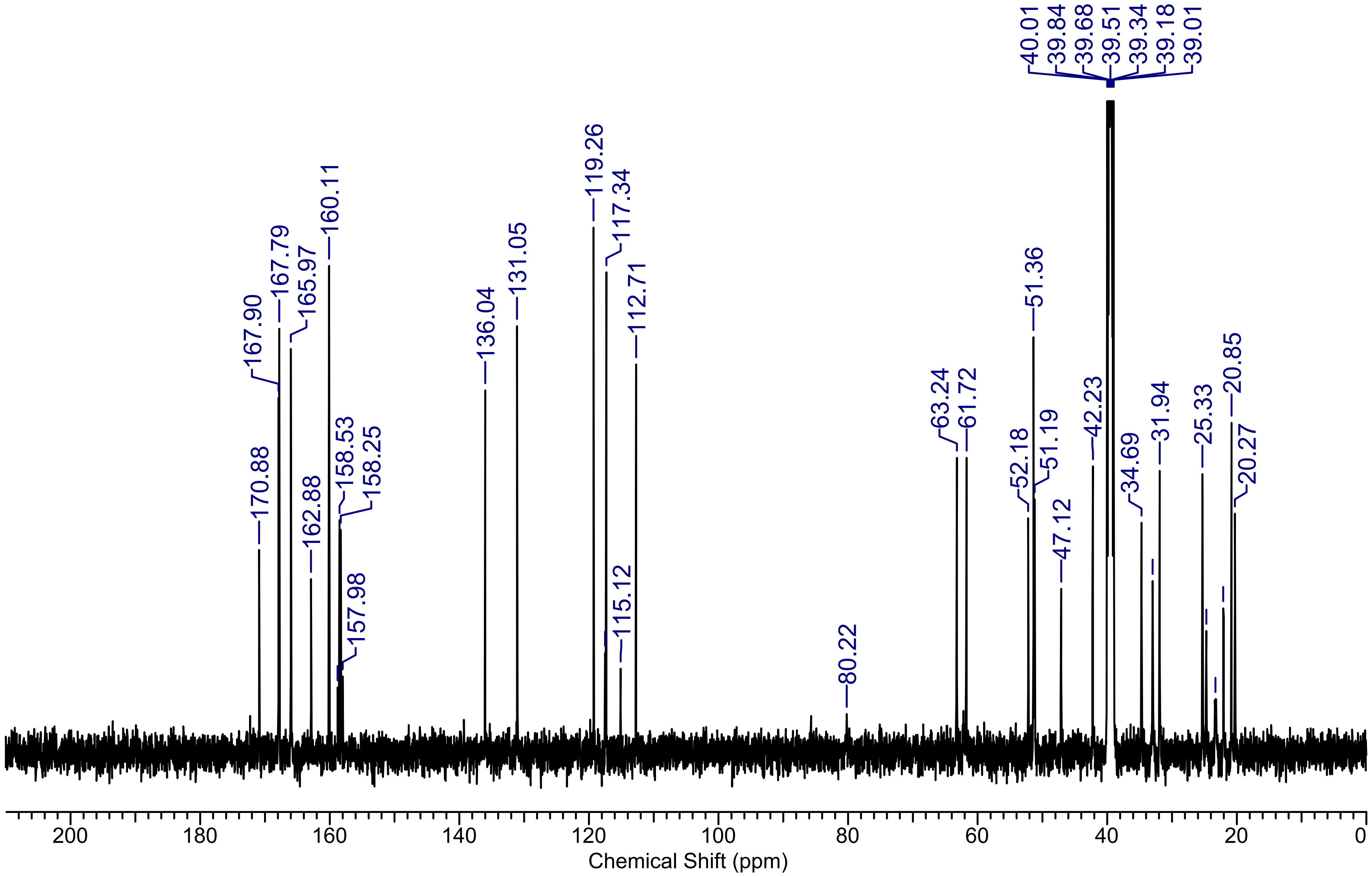

Figure S24. ^{13}C-NMR (125 MHz, dimethyl sulfoxide-d_6) spectrum of madurastatin H2 (**2**).

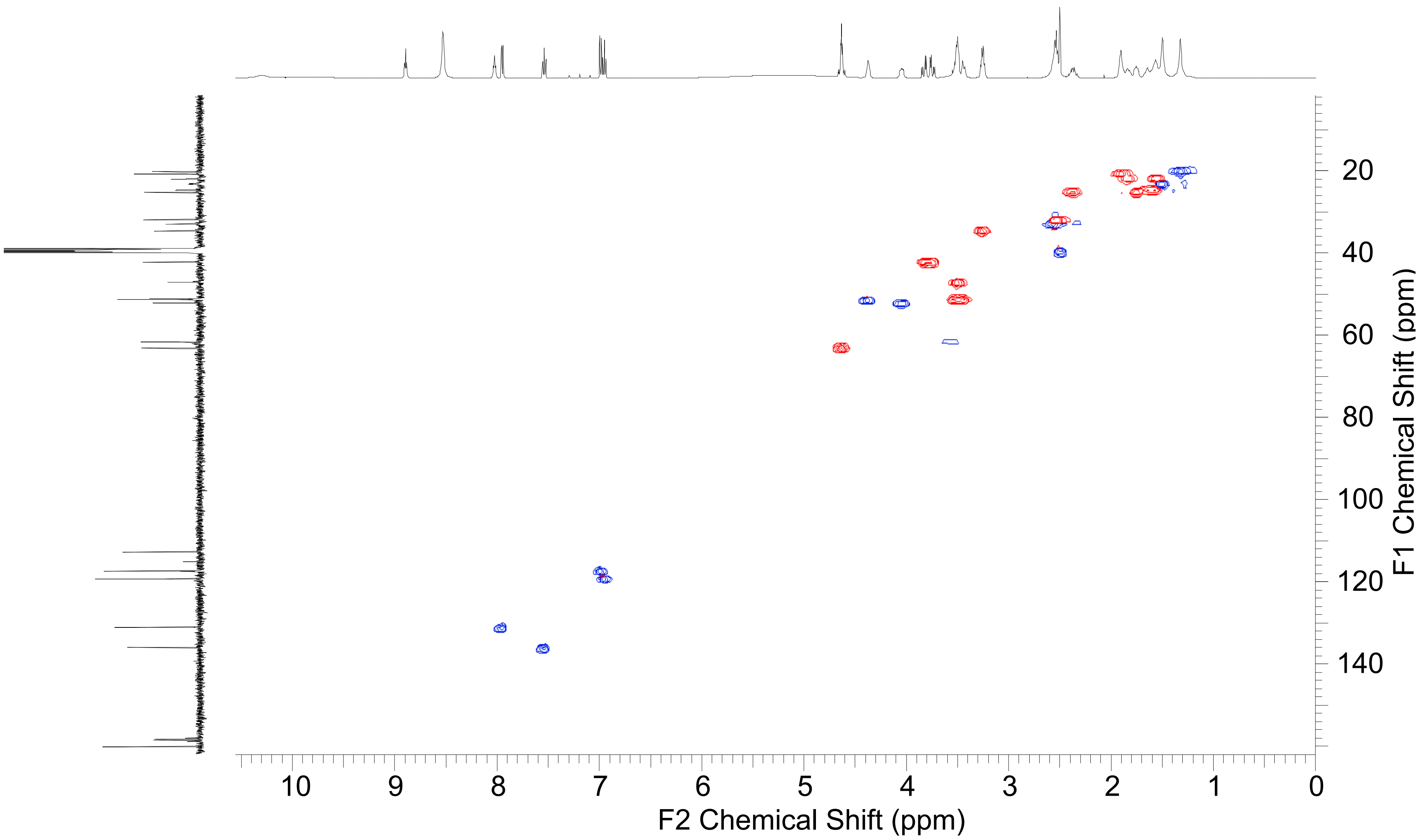

Figure S25. HSQC (dimethyl sulfoxide-d_6) spectrum of madurastatin H2 (**2**).

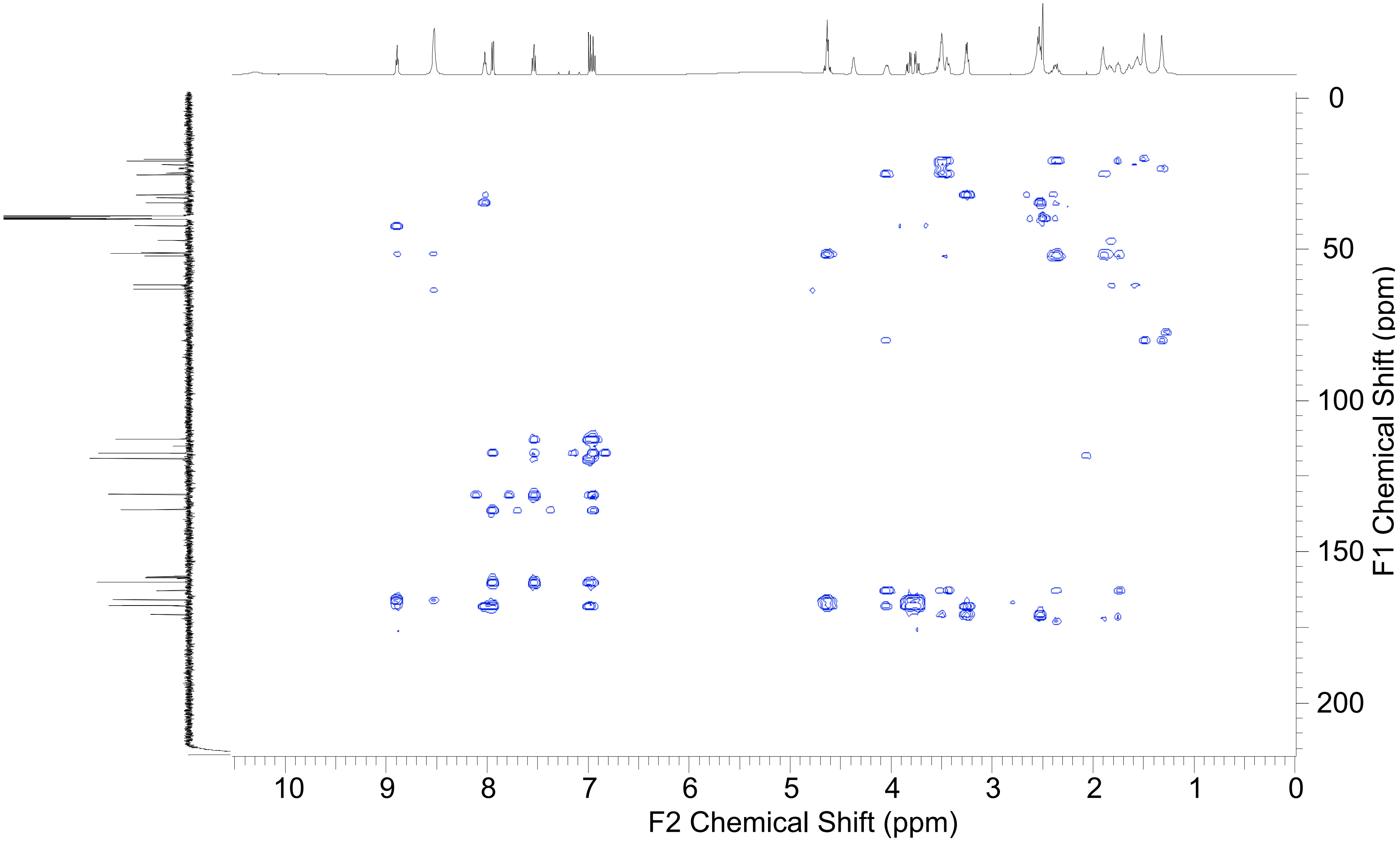

Figure S26. HMBC (dimethyl sulfoxide-d_6) spectrum of madurastatin H2 (**2**).

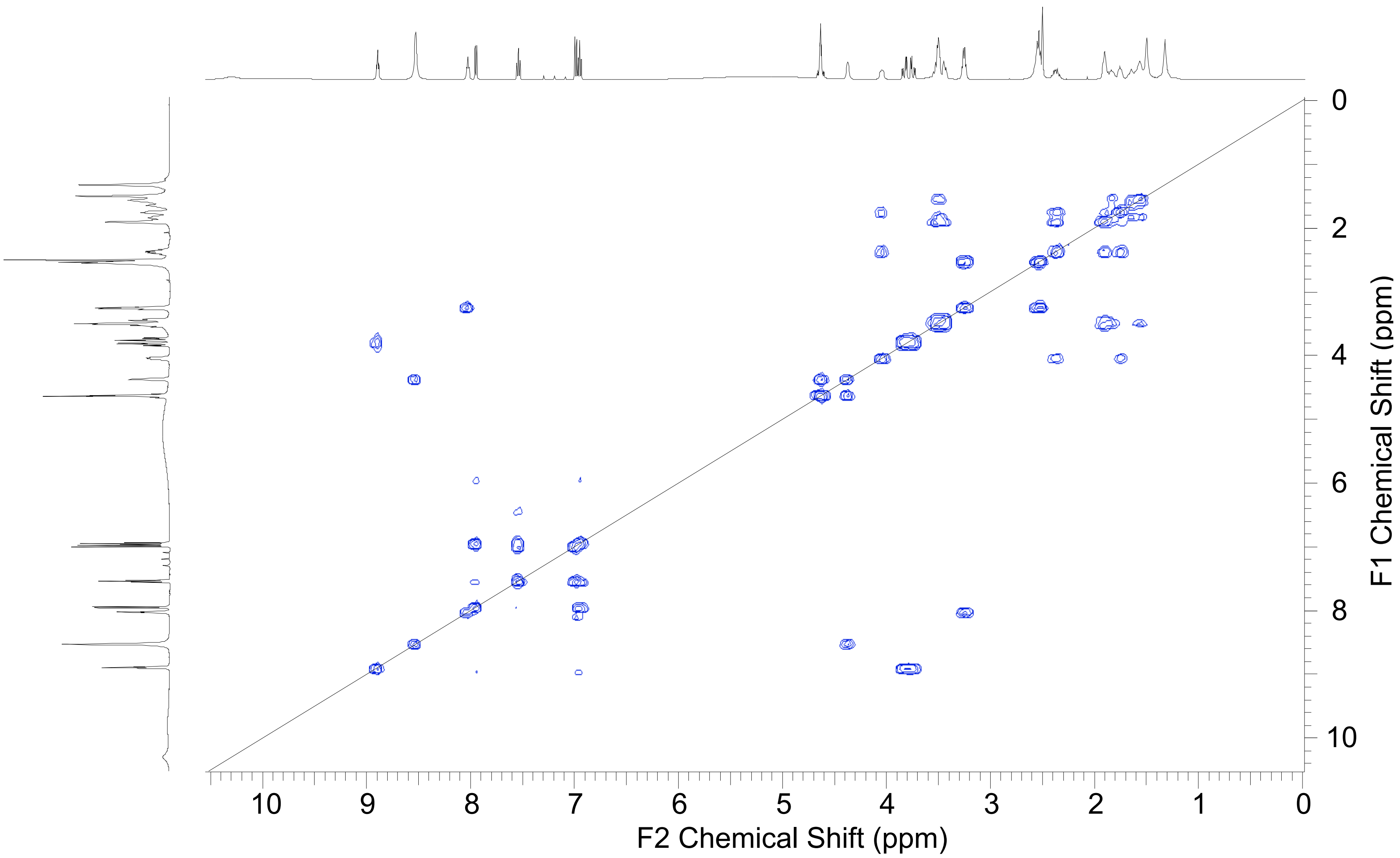

Figure S27. COSY (dimethyl sulfoxide-d_6) spectrum of madurastatin H2 (**2**).

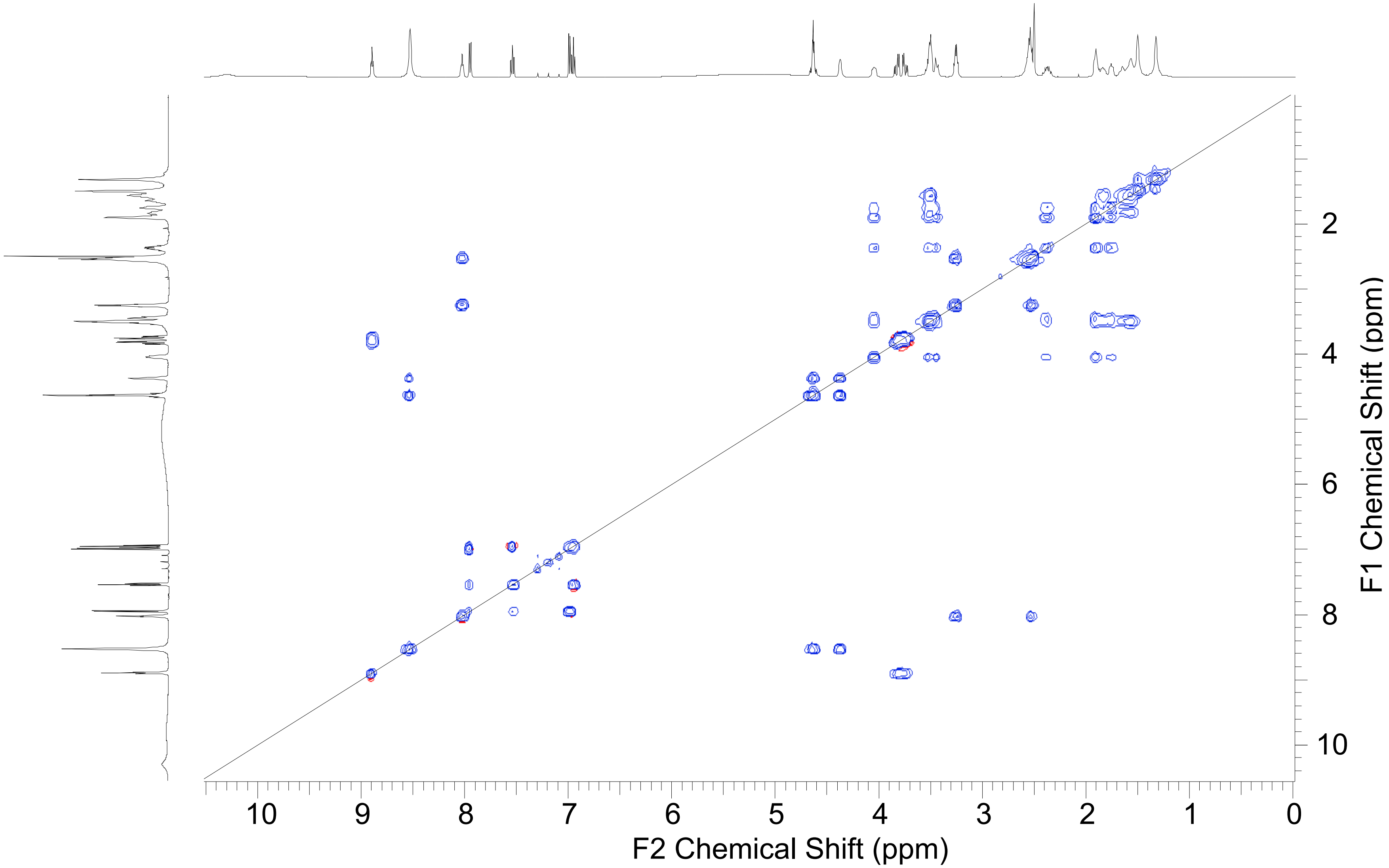

Figure S28. TOCSY (dimethyl sulfoxide-d_6) spectrum of madurastatin H2 (**2**).

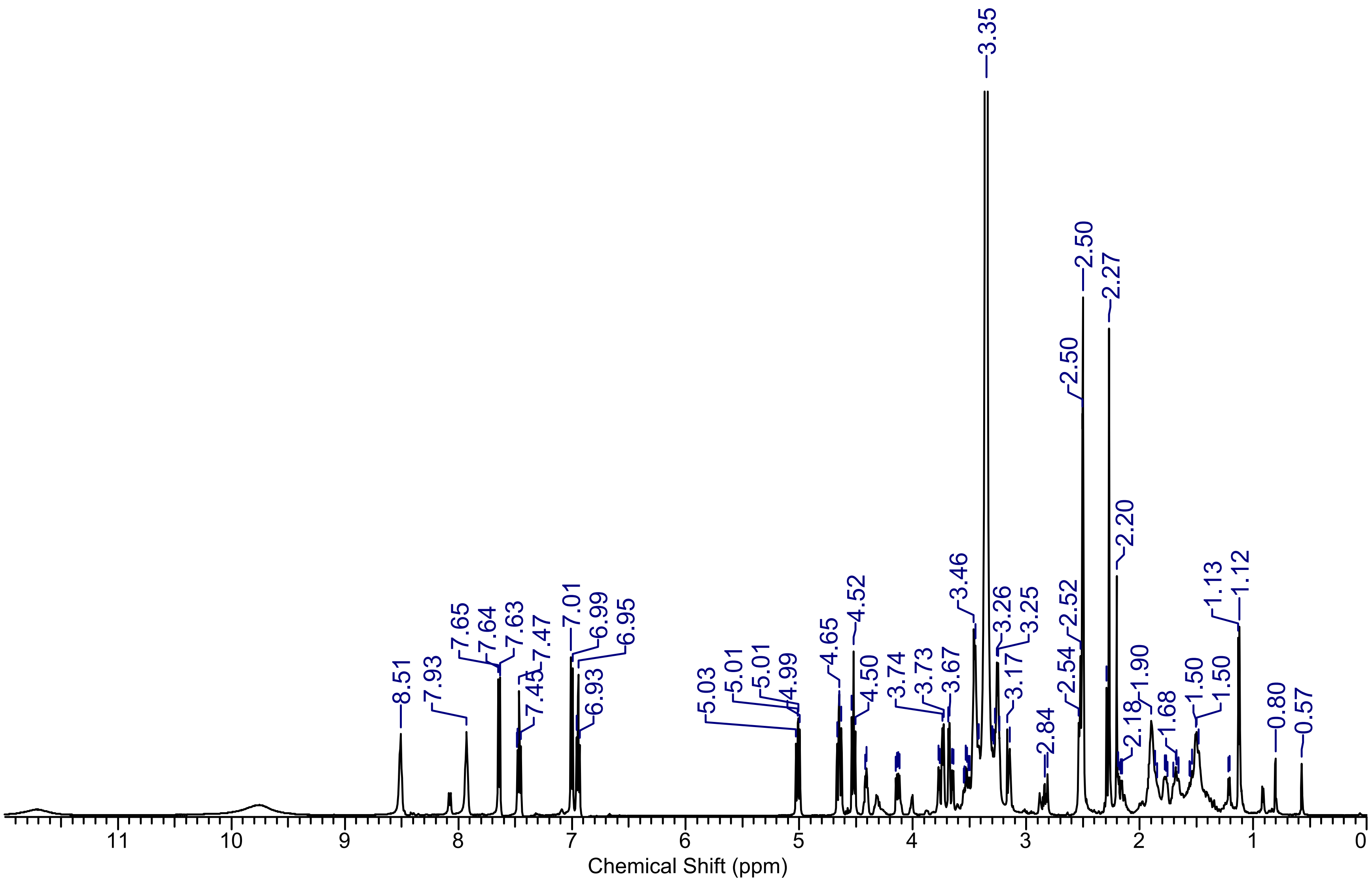

Figure S29. ^{1}H-NMR (500 MHz, dimethyl sulfoxide-d_6) spectrum of 33-*epi*-madurastatin D1 (**3**).

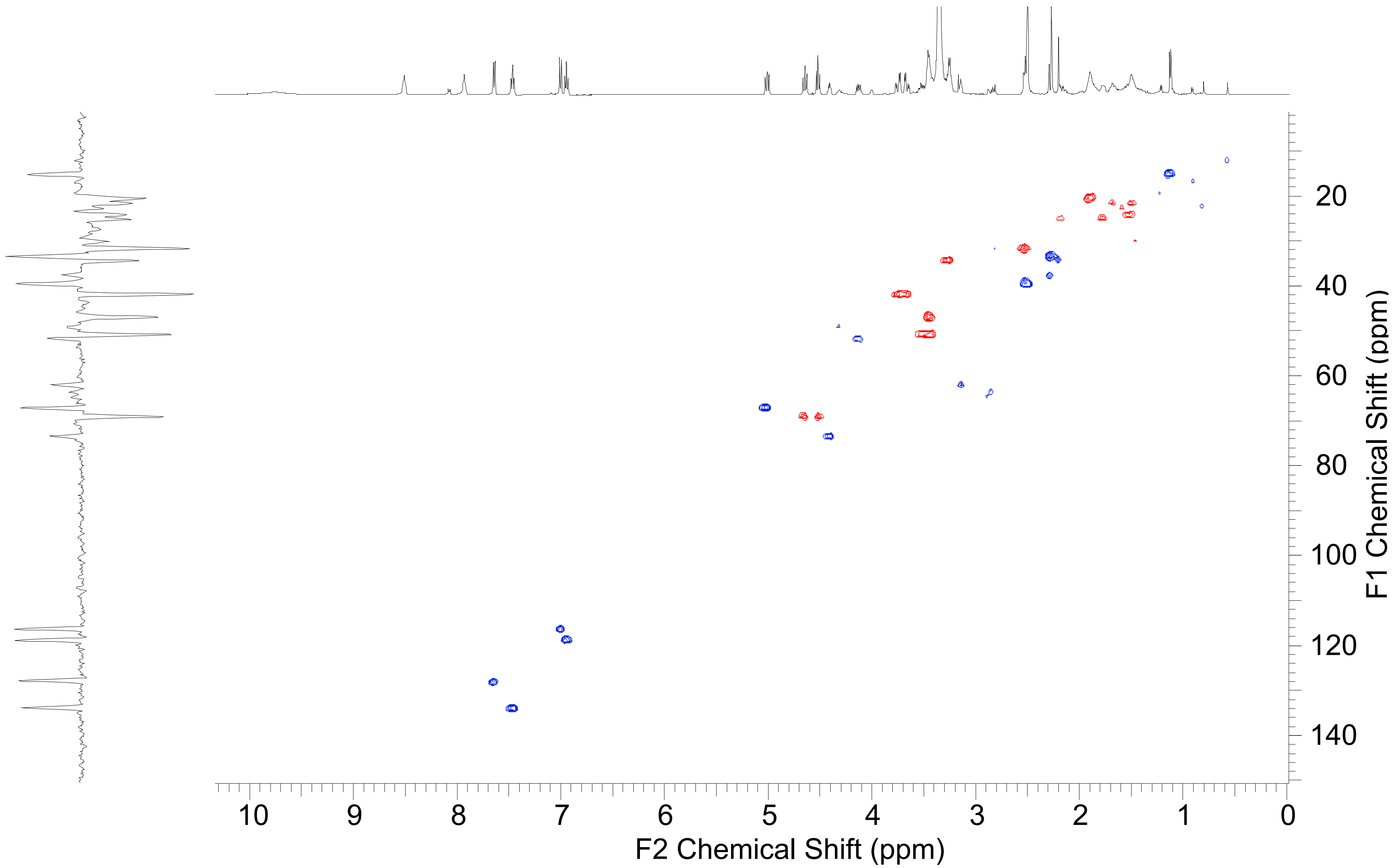

Figure S30. HSQC (dimethyl sulfoxide-*d*6) spectrum of 33-*epi*-madurastatin D1 (**3**).

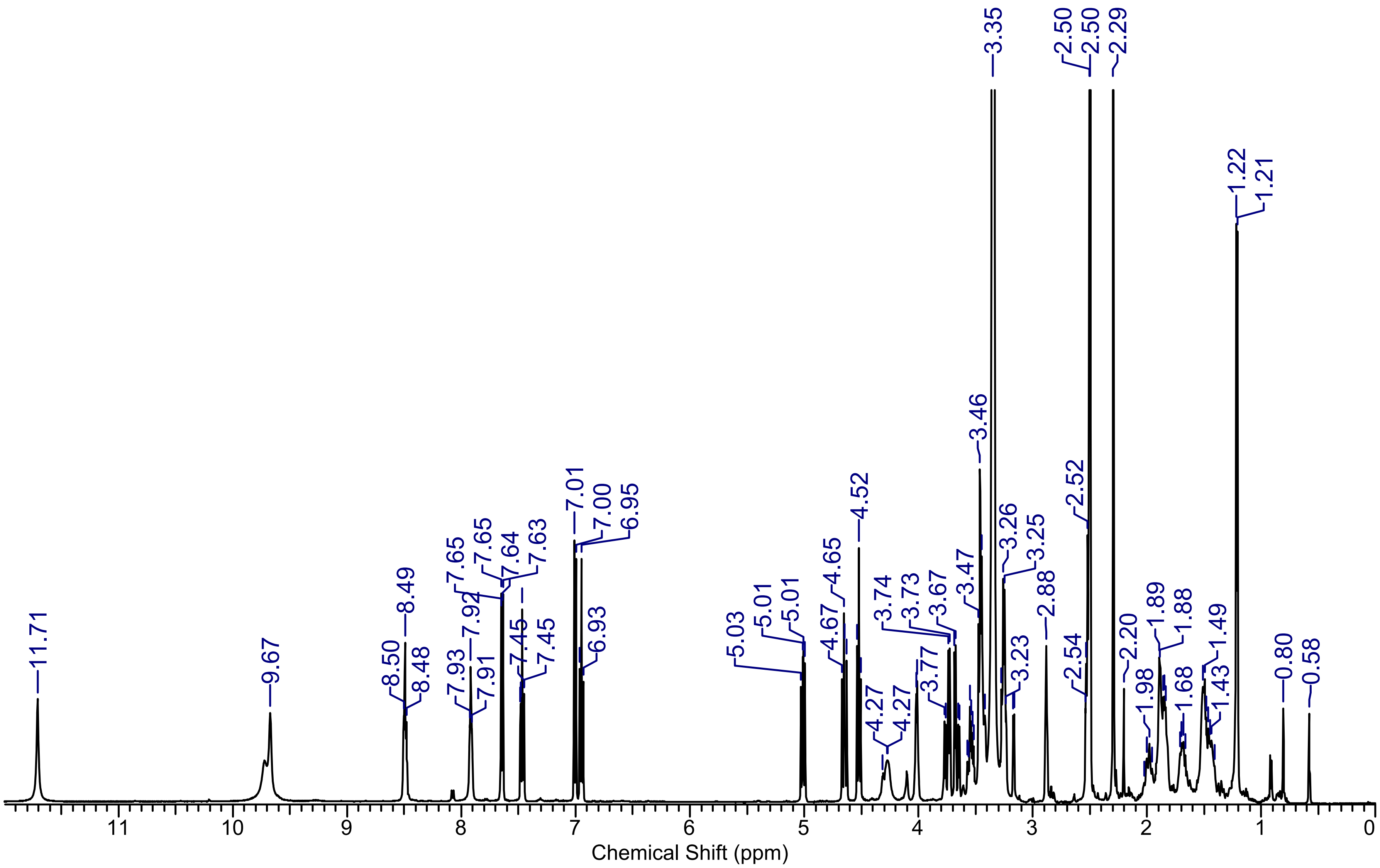

Figure S31. ^{1}H-NMR (500 MHz, dimethyl sulfoxide-d_6) spectrum of madurastatin D1 (**4**).

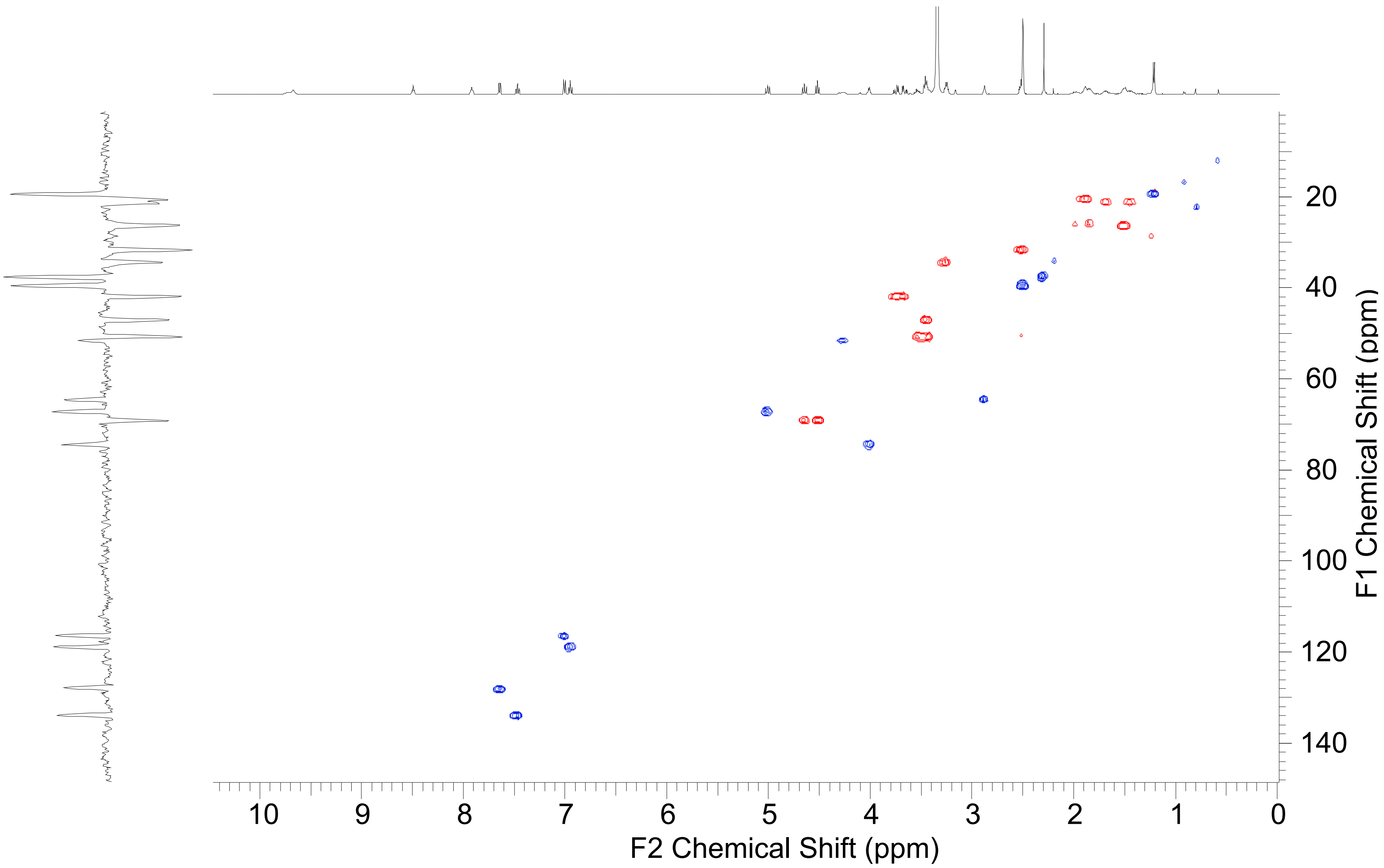

Figure S32. HSQC (dimethyl sulfoxide-d_6) spectrum of madurastatin D1 (**4**).

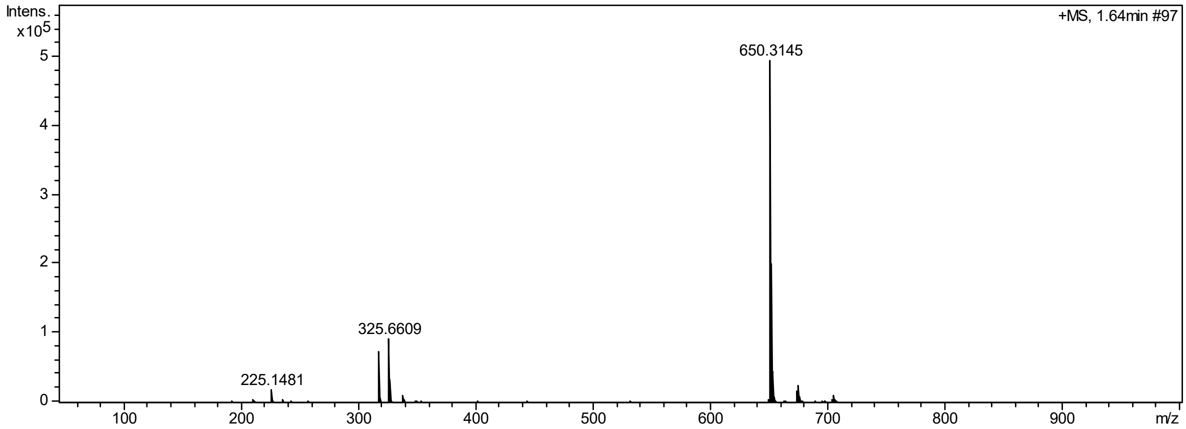

Figure S33. HRMS spectrum of madurastatin H2 (**2**).

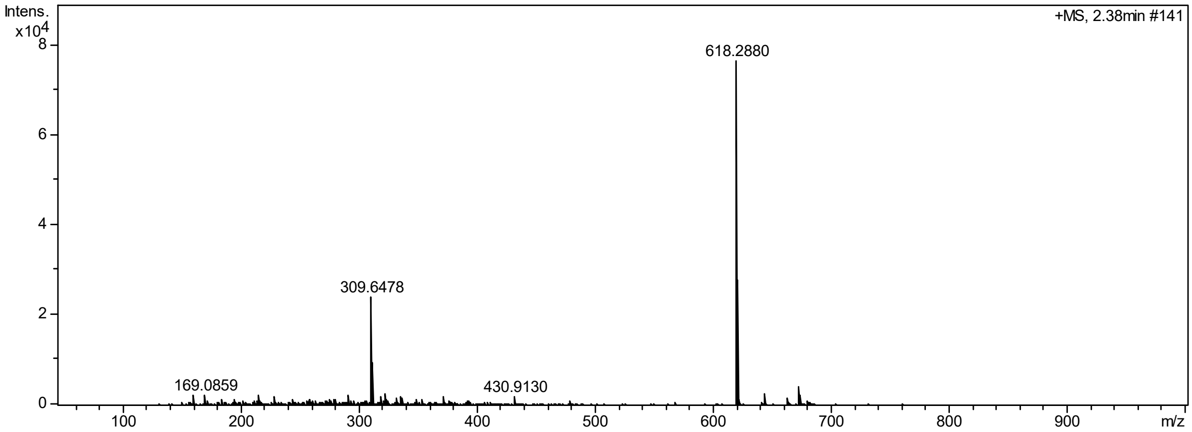

Figure S34. HRMS spectrum of 33-*epi*-madurastatin D1 (**3**).
